# Trends in the acceptability and prevalence of intimate partner violence: Evidence from Demographic and Health Surveys, 1999–2024

**DOI:** 10.1371/journal.pgph.0006628

**Published:** 2026-06-24

**Authors:** Irina Vartanova, Kimmo Eriksson, Pontus Strimling

**Affiliations:** 1 Institute for Futures Studies, Stockholm, Sweden; 2 Department of Women’s and Children’s Health, Uppsala University, Uppsala, Sweden; 3 Department of Business and Mathematics, Mälardalen University, Västerås, Sweden; 4 Institute for Analytical Sociology, Linköping University, Norrköping, Sweden; University of Washington Department of Global Health, UNITED STATES OF AMERICA

## Abstract

Intimate partner violence against women (IPVAW) is a major global public health problem. Social norms that accept IPVAW are a key correlate of its prevalence, and recent research has documented a widespread, long-term global decline in such acceptability. What societal changes underlie this normative shift and whether it is associated with a reduction in violence are not yet well understood. We analyzed Demographic and Health Surveys (DHS) data from 1999–2024, including responses from 1,920,105 women across 69 countries and 539,470 men across 60 countries. We calculated the annualized rate of change between the first and last available survey waves for each country. We tested the association between these country-level trends and longitudinal changes in the Human Development Index (HDI) and Gender Development Index (GDI) at a 4-year lag. We also examined whether trends in acceptability were correlated with trends in the reported prevalence of physical IPVAW. Acceptability of IPVAW declined in 94% of countries for women and 89% for men, with a mean annual decline of approximately 1.3 percentage points. Countries with faster increases in HDI tended to show faster declines in acceptability at a 4-year lag (women: *r* = -0.33; men: r = -0.54). Overall societal development (HDI) showed somewhat stronger associations with declining acceptability than gender-specific equity gains (GDI). Finally, countries with faster declines in acceptability tended to show faster reductions in physical IPVAW prevalence (r = 0.35). This study provides the first cross-national evidence that improvements in human development are associated with a global decline in the acceptability of IPVAW, and that this normative shift corresponds with reductions in experienced violence. These findings are consistent with a role for comprehensive development investments in education, health, and economic prosperity, though the design cannot establish that such investments cause reductions in violence against women.

## Background

Intimate partner violence against women (IPVAW) is a major global public health problem, affecting an estimated 641 million women worldwide [1,2]. It encompasses physical, sexual, and psychological violence, with severe and long-lasting health consequences including injury, depression, anxiety, post-traumatic stress disorder, and death [3]. Given its scale and impact, ending IPVAW is a central target of the United Nations Sustainable Development Goals (SDG 5.2).

The socio-ecological model [4] provides a framework for understanding how factors at individual, relationship, community, and societal (macrosystem) levels interact to influence violence risk. At the macrosystem level, social norms that justify IPVAW—such as the belief that a husband is entitled to physically discipline his wife—are consistently identified as a key correlate of its prevalence [5–7]. Both individual attitudes and community-level acceptability are strongly associated with IPVAW experience and perpetration [5,8–10]. Understanding how these norms are changing is therefore critical for prevention efforts.

Recent research has documented a widespread, long-term global decline in the acceptability of IPVAW. Throughout this paper, ‘acceptability of IPVAW’ refers to endorsement of the view that a husband is justified in hitting or beating his wife under at least one specified circumstance, as measured by items from the Demographic and Health Surveys (DHS), a long-running program of nationally representative household surveys conducted in low- and middle-income countries. Foundational work by Pierotti documented this decline during the first decade of the 21st century [11], and a comprehensive 2025 study by Bergenfeld and colleagues confirmed its durability: using DHS and Multiple Indicator Cluster Surveys (MICS) data from 83 countries until 2022, they found that acceptability declined significantly in most countries among both women and men [12]. However, two critical questions remain unresolved.

First, what societal changes underlie this normative shift? Modernization theory posits that economic development fosters value shifts toward greater gender equality and reduced tolerance of interpersonal violence [13,14]. This endogenous process of development-driven change is complementary to the role of international cultural diffusion of anti-violence norms emphasized by other scholars [11,15]. Cross-sectional evidence supports this relationship: countries with higher Human Development Index (HDI) scores exhibit lower acceptability of IPVAW [16], and associations exist between gross domestic product (GDP) per capita, urbanization, and lower domestic violence [17]. However, whether improvements in development over time correspond with declining acceptability remains unexplored.

Other scholars have emphasized exogenous explanations for normative change that operate independently of domestic economic development. Pierotti, analyzing DHS data from 26 countries, found that structural changes within countries—rising urbanization, education, and media access—could not account for the decline in IPVAW acceptability; she attributed the trend to the diffusion of a global cultural script condemning domestic violence, transmitted through international organizations, media, and education systems [11]. Swindle extended this by showing that mass media exposure is a primary conduit for normative diffusion, with media access predicting individual-level attitude change [15]. A parallel pathway operates through institutional mechanisms: True and Mintrom demonstrated that gender mainstreaming adoption across 157 countries was driven by transnational networks and participation in UN women’s conferences, not by domestic development levels [18]. Htun and Weldon, analyzing 70 countries over four decades, found that autonomous feminist mobilization—not national wealth or women’s political representation—was the strongest predictor of progressive policy on violence against women [19]. These endogenous and exogenous mechanisms are not mutually exclusive; countries experiencing development improvements may simultaneously face greater exposure to international human rights discourses. Development may facilitate normative diffusion by expanding education, media access, and civic capacity—making it difficult to disentangle whether norm change reflects endogenous value shifts or increased reception of exogenous global scripts. Our analysis documents associations between development trends and normative change but cannot adjudicate between these complementary explanations.

This raises an important question: is the observed normative shift associated with general societal progress (rising living standards for all), or specifically with improvements in women’s status relative to men? While HDI captures overall development, the Gender Development Index (GDI) measures gender gaps in health, education, and economic resources. Within Heise’s socio-ecological framework, gender inequality operates as a fundamental macrosystem-level driver of violence [4]. We therefore examine both overall development (HDI) and gender-equitable development (GDI) to understand which dimensions of societal change are most strongly associated with normative shifts.

Second, does normative change translate into reduced violence? As Bergenfeld et al. note, this “is far from straightforward” [12]. A 2023 study in *The Lancet Global Health* found an overall decline in physical and/or sexual IPVAW prevalence across 53 countries between 2000 and 2021, linking this decline to indicators of women’s empowerment [20]. Yet the relationship between attitudinal shifts and prevalence trends has not been directly examined. Understanding whether declining acceptability corresponds with declining violence is essential for assessing whether norm-change interventions may contribute to reducing IPVAW.

**Fig 1:**
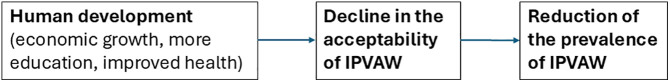
Analytic framework. Arrows indicate the hypothesized relationships tested in this study: human development (HDI, GDI) is associated with trends in the acceptability of IPVAW, and trends in acceptability are associated with trends in prevalence.

This study addresses both questions by analyzing data from the DHS, a series of nationally representative household surveys conducted across low- and middle-income countries, from 1999–2024, including responses from 1,920,105 women across 69 countries and 539,470 men across 60 countries. We test whether country-level trends in IPVAW acceptability are associated with earlier trends in HDI and GDI, examining temporal lags to identify when development improvements most strongly relate to norm change. We then examine whether trends in acceptability correlate with trends in the reported prevalence of physical IPVAW. (Fig 1, in the Methods section, illustrates the specific relationships tested in this study.) Within the broader socio-ecological framework of IPVAW, we focus on macrosystem-level pathways: whether improvements in human development correspond with declining acceptability of IPVAW, and whether declining acceptability corresponds with reduced violence prevalence. While correlational evidence cannot establish causality, correlations between trends provide a stronger test of hypothesized relationships than correlations in cross-sectional data. Cross-sectional correlations confound the hypothesized effect with all stable between-country differences (e.g., geography, colonial history, cultural heritage). Correlations between within-country changes over time control for these time-invariant factors, isolating the association between temporal co-movements [21].

By examining both overall development (HDI) and gender-equitable development (GDI), this analysis clarifies whether and how global progress in human development is translating into gender-equitable social norms and, ultimately, safer lives for women.

## Methods

### Data sources

This study is a secondary analysis of data from the DHS Program [22], publicly available at https://dhsprogram.com/. We used the rdhs R package to identify and download relevant datasets [23]. The DHS provides comprehensive and comparable population-level data across many countries and time points. While dedicated violence-against-women surveys using the World Health Organization (WHO) Multi-country Study methodology are considered the gold standard for measuring IPVAW, the DHS domestic violence module offers the broad geographic and temporal coverage that enables the large-scale trend analysis presented here.

We compiled all available DHS datasets containing the domestic violence module released between 1999 and 2024. To ensure comparability across surveys and time, our analysis is restricted to participants (both women and men) aged 15–49 years who are or have been married, and who provided information on IPVAW acceptability or prevalence. This comprises responses from 1,920,105 women in 69 countries and 539,470 men in 60 countries.

For the primary trend analysis, only countries with at least two surveys collecting data on IPVAW acceptability or prevalence were included. For cross-sectional analyses, such as correlating initial levels of acceptability with the Human Development Index, countries that participated only once were also included. A complete list of countries, survey years, and sample sizes is available in Table A in S1 Appendix.

### Measures

#### Acceptability of IPVAW.

DHS measures attitudes toward IPVAW by asking respondents whether they agree that a husband is justified in hitting or beating his wife for each of several specific reasons: burning food, arguing with him, going out without telling him, neglecting the children, and refusing to have sexual intercourse with him. We focus on countries where all five contexts were included in the survey. Following established literature [6,12], a respondent was coded as accepting IPVAW if they did not reject that a husband is justified in hitting or beating his wife for at least one of these five reasons. We calculated the weighted percentage of respondents accepting IPVAW for each country-year.

It is important to note that the DHS does not include questions about justifying violence in response to suspected or actual infidelity. Research using the World Health Organization (WHO) Multi-country Study methodology has found that infidelity is consistently the most widely accepted justification for violence in many contexts [24]. Thus, our DHS-based estimates likely represent a conservative (lower-bound) measure of total IPVAW acceptability.

#### Prevalence of physical IPVAW.

The DHS Domestic Violence module is an optional component included in some but not all country surveys. It is administered to a randomly selected subsample of women in each household and measures various forms of violence, including intimate partner violence (for ever-partnered women) and non-partner violence.

We measured women’s experiences of physical IPVAW through questions directed to these randomly selected subsamples of women who ever lived with a partner. To align conceptually with the acceptability measure (whether a husband is justified in ‘hitting or beating his wife’), we focused on physical violence. A respondent was coded as having experienced physical IPVAW if she reported that a partner had pushed her, shook her, threw something at her, slapped her, twisted her arm or pulled her hair, punched her, or hit her with something in the last 12 months. We excluded two items about more extreme violence—being strangled or burnt, and being threatened with a weapon—because they are conceptually distinct from the ‘hitting or beating’ specified in the survey’s social norms questions.

The prevalence of physical IPVAW in a country-year is the weighted percentage of women reporting such violence in the past year.

#### Human development measures.

We obtained Human Development Index (HDI) data directly from the United Nations Development Programme (UNDP) Human Development Reports database [25]. The HDI is calculated as the geometric mean of normalized country indices for three key dimensions: health (life expectancy at birth), education (mean years of schooling for adults and expected years of schooling for children), and economic prosperity (logarithm of gross national income per capita). The UNDP publishes new HDI measures annually.

To distinguish between the roles of overall development versus gender-equitable development, we also analyzed the Gender Development Index (GDI). The GDI measures the ratio of female-to-male achievement in the same three HDI dimensions. A GDI value closer to 1 indicates greater gender parity, while values further from 1 indicate larger gender gaps. Improvements in GDI therefore reflect the narrowing of gender inequalities in health, education, and economic resources. GDI data were also obtained from UNDP.

This parallel analysis allows us to test whether normative change is associated primarily with rising living standards that benefit entire populations (captured by HDI) or specifically with women’s gains relative to men (captured by GDI). Fig 1 provides an overview of the relationships tested.

#### Covariates for robustness analyses.

For supplementary robustness analyses, we obtained four time-varying country-level covariates. Net official development assistance (ODA) received as a percentage of gross national income (GNI), the percentage of seats held by women in national parliaments, and trade openness (the sum of exports and imports as a percentage of gross domestic product) were obtained from the World Bank World Development Indicators [26]. Armed-conflict intensity was coded as the country-year maximum state-based intensity level from the UCDP/PRIO Armed Conflict Dataset version 25.1 (0 = no active conflict, 1 = minor, 2 = war) [27].

### Statistical analysis

All country-level percentages for the prevalence and acceptability of IPVAW were calculated using the individual sample weights provided in the DHS datasets to ensure national representativeness. For acceptability data, we used standard DHS sample weights (v005). For prevalence data, we used the domestic violence-specific sampling weights (dv005), which adjust for both household selection probability and the within-household random selection of DV module respondents.

#### Estimating temporal trends.

Throughout this paper, a ‘trend’ refers to the estimated annual rate of change in a variable over the study period for a given country, measured in percentage points per year. To estimate the temporal trend for each country, we calculated the change in percentage points per year as the difference between the last and first observed values divided by the number of years between observations: (*Y*_last_ − *Y*_first_)/ (*t*_last_ − *t*_first_). This two-point estimator directly measures the observed change rate across the study period for each country. The standard error (SE) for this rate was computed by propagating the sampling uncertainty of the two proportion estimates: SE = √(SE_first_^2^ + SE_last_^2^)/ (*t*_last_ − *t*_first_), where SE_*t*_ = √[*p*_t_(1 − *p*_t_)/*n*_t_]. In this formula, *p*_t_ represents the proportion (of acceptability or prevalence) and *n*_t_ the sample size at time t, while the denominator *t*_last_ − *t*_first_ accounts for the length of the observation period to produce a standardized annual estimate. This approach allows for the construction of 95% confidence intervals (Rate ±1.96 SE) that directly reflect the inherent sampling noise of the underlying DHS surveys. Importantly, any measurement error in these country-specific trend estimates acts to attenuate correlations toward zero in subsequent analyses (classical errors-in-variables bias); our correlational findings are therefore conservative.

While countries vary in the number of survey waves (range: 2–9, median: 4), this two-point approach focuses on the total observed change rather than fitting a linear trend to potentially non-linear intermediate data. This method provides unbiased estimates of the annualized rate of change between first and last observations, with confidence intervals that directly reflect sampling uncertainty. However, the results we present are virtually identical if we instead use ordinary least squares (OLS) estimates based on all points in time with available data (as shown in our robustness analysis).

This approach directly estimates additive changes in acceptability levels over time. It differs from the recent comprehensive study by Bergenfeld et al. [12], who used generalized linear models with a log link to model trends. While their figures refer to “annual percentage-point change,” log-link models actually estimate relative (multiplicative) changes. The two approaches are complementary and yield consistent results. Our percentage-point approach offers several advantages: (1) results are directly interpretable as absolute changes in acceptability, (2) trends can be directly compared to baseline prevalence levels, and (3) the relationship between acceptability trends and prevalence trends (both measured in percentage points) is more straightforward to interpret.

#### Association with human development.

To examine the relationship between trends and living standards, we used Pearson correlation coefficients. If increases in HDI are associated with subsequent decreases in IPVAW acceptability, this predicts that changes in HDI will be correlated with later changes in acceptability. However, a priori, the lag between development improvements and normative change is theoretically uncertain, and no prior empirical work specifies an expected duration. We therefore correlated country-level trends in acceptability with the corresponding change in the country’s HDI over equally long periods but occurring a few years earlier, using lags ranging from 1 to 10 years (for countries where the lagged start year preceded the earliest available HDI record, the period was truncated to begin at the first available data point, so the HDI change period may be shorter than the acceptability trend period). We similarly correlated initial baseline levels of acceptability (at the first point of measurement) with baseline HDI.

The same analysis was repeated using GDI in place of HDI to test whether gender-equitable development shows different associations with normative change than overall development. For HDI and GDI trends, we used OLS linear regression rather than the two-point estimator. With longer lags, the required start year for estimating development change may predate the earliest available HDI and GDI data in some countries; OLS regression accommodates this by fitting trends to all available data points within the specified period.

#### Relationship between declining acceptability and declining prevalence.

This same correlation method was used to assess the relationship between trends in acceptability and trends in prevalence. We reestimated time trends over the period for which data on both prevalence and acceptability were available. Where acceptability data were available for both men and women, their trends were averaged; in five countries that lacked trend data for men, only women’s acceptability trends were used.

### Hierarchical longitudinal robustness analysis

As a robustness check addressing concerns about shared-trend confounding, we fit a hierarchical longitudinal (multilevel) model to the wave-level acceptability data. Each country-year DHS wave enters as an observation, inverse-variance weighted by its binomial sampling variance (n/ [p(1 − p)], normalised to mean 1 within sex). The model includes country-specific random intercepts and random linear time trends, plus a fixed global year term, so that each country’s idiosyncratic trajectory is absorbed and the within-country HDI coefficient is identified by deviations from that trajectory. Time-varying predictors — HDI (at the 4-year lag used in the main analysis) and four supplementary confounders — are decomposed into country-mean (between-country) and deviation-from-country-mean (within-country) components following Bell & Jones (2015). The outcome and all time-varying predictors are standardised per sex (pooled SD), so coefficients are standardised β. Three nested specifications are reported (Table B in S1 Appendix): HDI alone; plus plausibly-exogenous lagged controls (UCDP/PRIO armed-conflict intensity; ODA as % of GNI); plus partly-endogenous lagged controls (trade openness; women’s parliamentary representation). Models were fit in R with lme4 and lmerTest using REML and the bobyqa optimiser, with Satterthwaite degrees of freedom.

All data processing and analysis were conducted in R version 4.5.3 [28].

### Ethical considerations

This study is a secondary analysis of publicly available, anonymized data from the DHS Program. All original DHS surveys are approved by the Institutional Review Board (IRB) of ICF and by a local IRB in the host country, and informed consent is obtained from all respondents before participation.

## Results

### Widespread decline in the acceptability and prevalence of IPVAW

Our analysis reveals a widespread and significant decline in the acceptability of IPVAW across the countries studied. For the acceptability of IPVAW among women, the estimated time trends were negative in 44 out of 47 countries (94%), with a mean decrease of 1.30 percentage points per year. This global decline was not confined to a specific period; for the 21 countries included in Pierotti’s original study [11], the decrease in the acceptability of IPVAW among women continued robustly: the mean decrease was 1.82 percentage points per year in 1999–2010 and 0.91 percentage points per year in 2011–2024. Country-level trend estimates for acceptability and prevalence are reported in Table C in S1 Appendix.

Results for time trends in the acceptability of IPVAW among men were similar. The trends were negative in 33 out of 37 countries (89%), with a mean decrease of 1.31 percentage points per year. These rates are comparable to those reported by Bergenfeld et al. [12], who found declining acceptability in 94% and 89% of countries for women and men respectively.

**Fig 2:**
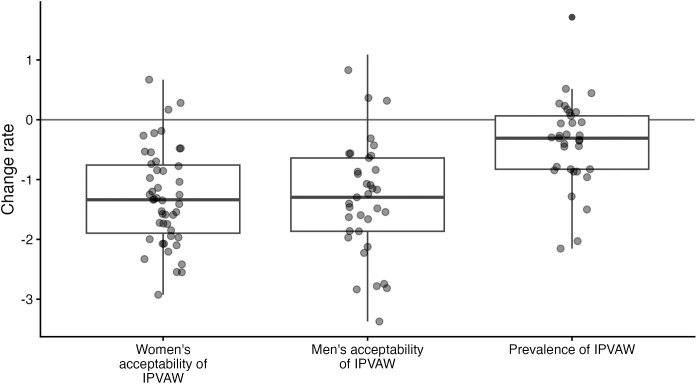
Estimated time trends for the acceptability and prevalence of IPVAW. The figure shows box plots of estimated time trends, in percentage points per year, for (from left to right) the acceptability of IPVAW among women in 47 countries, the acceptability of IPVAW among men in 37 countries, and the prevalence of IPVAW in 33 countries.

Fig 2 visually represents these downward trends in the acceptability of IPVAW among women and men. The prevalence analysis showed that 73% of countries (24 out of 33) experienced a reduction in reported physical IPVAW.

### Consistency of trends in the acceptability of IPVAW across contexts

Within each country, the trend in acceptability of IPVAW was highly consistent across different contexts (burning food, arguing, neglecting children, going out without telling, refusing sex), as shown by the strong intercorrelations and similarity of average change rates reported in Table 1.

**Table 1:** Consistency across contexts in country-specific time trends in the acceptability of IPVAW among women.

	Argue	Burn food	Neglect children	Go out w/o telling	Refuse sex
Argue	*-0.75*				
Burn food	0.50	*-0.62*			
Neglect children	0.63	0.66	*-1.10*		
Go out w/o telling	0.62	0.69	0.83	*-1.04*	
Refuse sex	0.62	0.59	0.55	0.72	*-0.71*

*Note.* Entries are Pearson correlations between the estimated time trends for the acceptability of IPVAW among women in different contexts. The italicized numbers on the diagonal are the average trend in percentage points per year for that context. Based on n = 47 countries.

### Consistency of trends in the acceptability of IPVAW across genders

The correlation between the rate of decline in acceptability among women and the rate of decline among men within the same country is r = 0.58, 95% CI [0.31, 0.76], n = 37 countries. This moderate-to-strong correlation indicates substantial consistency in how acceptability is changing across genders within countries, though not complete synchrony. While women and men are moving in similar directions at similar rates within each society, the imperfect correlation indicates that gender-specific factors also influence the pace of change.

### Association with human development and gender equity

We first tested whether improvements in the Human Development Index (HDI) correspond with declining acceptability of IPVAW. We found a strong cross-sectional relationship: countries with higher HDI at baseline showed significantly lower acceptability among both women (r = -0.67, 95% CI [-0.78, -0.51], n = 68) and men (r = -0.35, 95% CI [-0.56, -0.11], n = 60).

**Fig 3:**
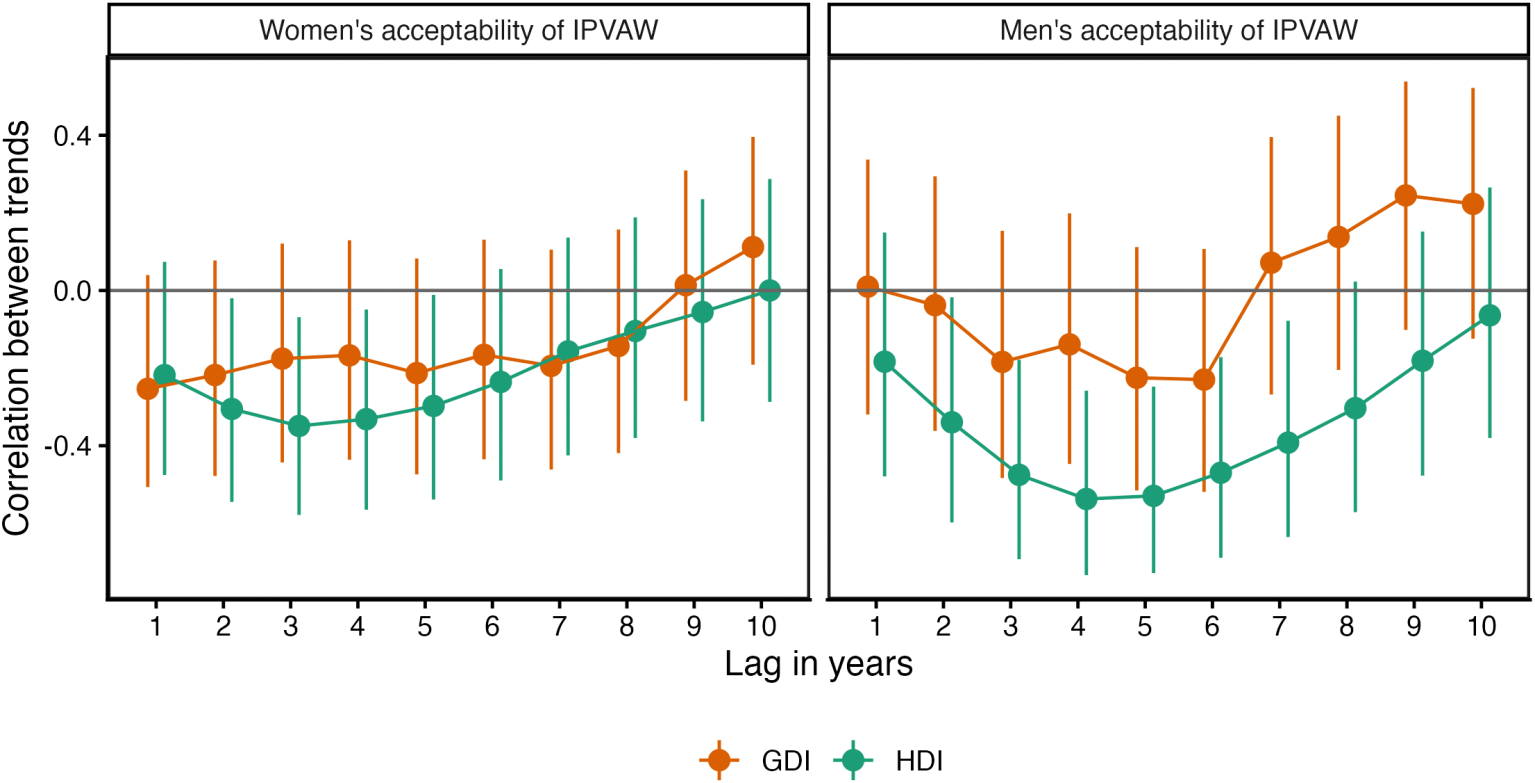
Correlation between development and acceptability trends across different time lags. Each line shows the correlation between change in development (HDI in green, GDI in orange) and change in acceptability of IPVAW, where the development change precedes the acceptability change by 1-10 years. Left panel: acceptability among women. Right panel: acceptability among men. Error bars represent 95% confidence intervals.

We also found that the pace of HDI improvement predicted the pace of attitudinal change several years later. Testing temporal lags from 1 to 10 years, we observed a U-shaped pattern with negative correlations strongest at moderate lags (Fig 3). Using a four-year lag, countries with faster HDI growth showed faster declines in acceptability among both women (r = -0.33, 95% CI [-0.56, -0.05], n = 47) and men (r = -0.54, 95% CI [-0.73, -0.26], n = 37).

To test whether this relationship reflects general societal progress or specifically the closing of gender gaps, we repeated the analysis using the Gender Development Index (GDI). As Fig 3 illustrates, improvements in gender equity consistently showed weaker associations with declining acceptability than overall HDI improvements across all lag periods tested. While the difference was modest at each individual lag, the pattern was consistent: HDI improvements showed stronger associations with declining acceptability than GDI improvements.

### Relationship between declining acceptability and declining prevalence

Acceptability trends (averaged across men and women except in five countries that lack trend data for men) show a positive correlation with trends in the prevalence of IPVAW (r = 0.35, 95% CI [-0.00, 0.62], n = 32).

**Fig 4:**
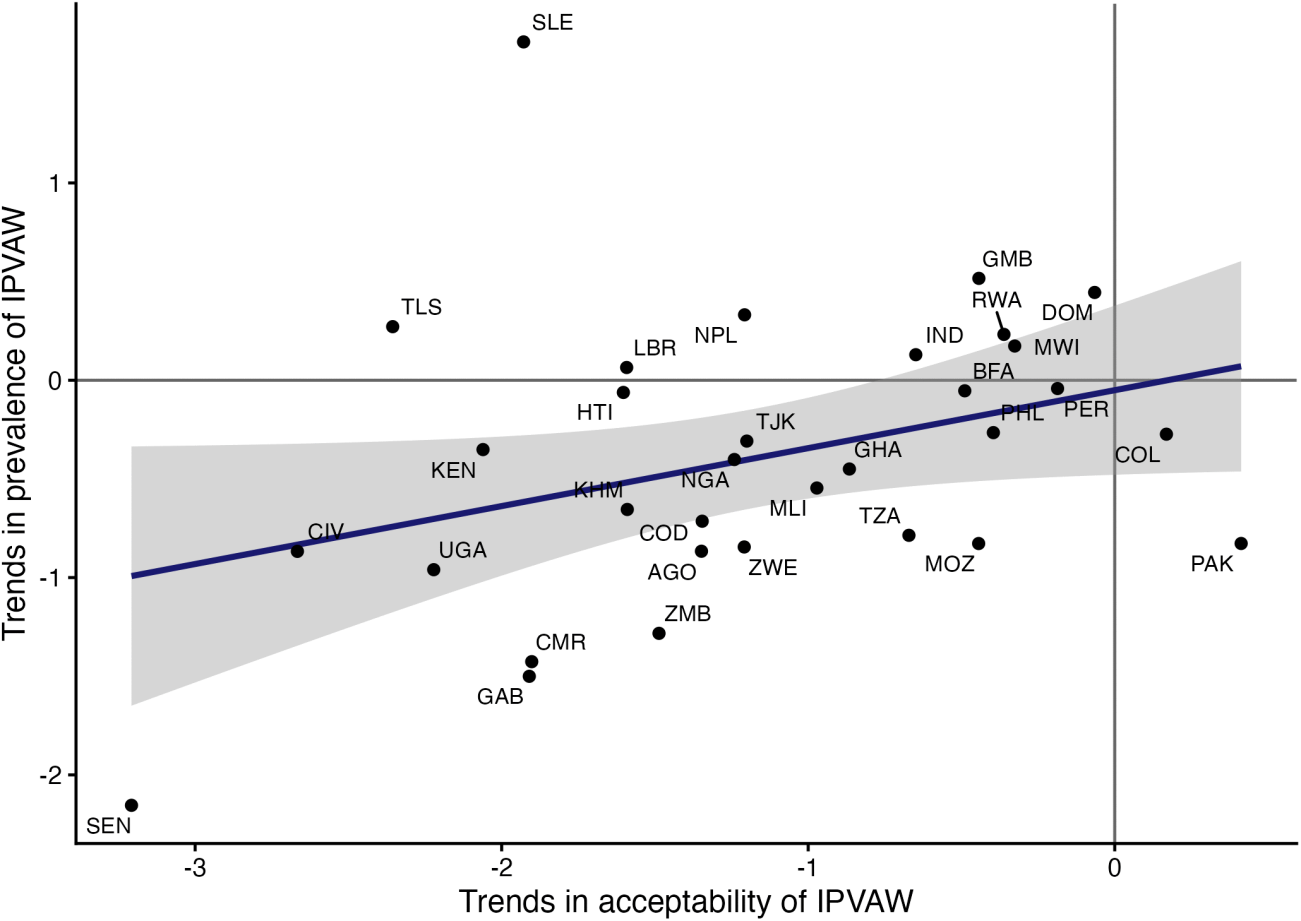
Scatterplot of estimated time trends for the prevalence of IPVAW (y-axis) plotted against time trends in acceptability of IPVAW (averaged between men and women). The unit on both axes is percentage points per year. Labels are ISO country codes.

Visual inspection of Fig 4 reveals Sierra Leone (SLE) as an outlier, the only country with a strong increase in the prevalence of IPVAW. If this outlier is excluded, the observed correlation strengthens to r = 0.51, 95% CI [0.20, 0.73], n = 31, suggesting that our estimate of the relationship between acceptability trends and prevalence trends is conservative. These correlations are unchanged if countries that lack trend data for men are excluded.

### Robustness analyses

As a robustness check, we re-estimated all country-level trends using ordinary least squares (OLS) regression fitted to all available survey waves, rather than the two-point estimates used in the main analysis. OLS slopes were highly correlated with the two-point estimates across all measures (Fig A in S1 Appendix), and re-running the key analyses with OLS-derived trends produced virtually identical lag profiles (Fig B in S1 Appendix), acceptability–prevalence associations (Fig C in S1 Appendix), and summary statistics (Table D in S1 Appendix). This consistency indicates that our findings are not sensitive to the choice of trend estimation method.

To further examine the robustness of the HDI-acceptability association, we conducted two supporting analyses. First, a falsification test confirmed temporal ordering: lag correlations (HDI changes preceding acceptability changes) were negative, whereas lead correlations (HDI changes following acceptability changes) were near zero (Fig D in S1 Appendix). Second, we fit a multilevel longitudinal model to the wave-level data with a series of control variables (Table D in S1 Appendix). Each country-year proportion enters as an observation, inverse-variance weighted by its binomial sampling variance; country-specific random intercepts and random linear time trends absorb country-idiosyncratic trajectories, while a fixed global year term absorbs the common secular trend. Time-varying predictors are decomposed following Bell & Jones (2015) into country-mean (between) and deviation-from-country-mean (within) components. Outcome and all time-varying predictors are standardised, so coefficients are standardised β. The within-country HDI coefficient — which is identified by deviations from each country’s own trajectory — is the quantity of substantive interest. Three nested specifications were fit separately for women (W1–W3) and men (M1–M3). With HDI alone, the within-country standardised β is −0.49 (95% CI [−0.88, −0.10], p = .014) for women and −1.04 [−1.51, −0.58], p < .001 for men. Adding plausibly-exogenous lagged controls (armed-conflict intensity; ODA as % of GNI) moves the coefficients to −0.56 (W2; p = .006) and −0.91 (M2; p < .001); further adding partly-endogenous lagged controls (trade openness; women’s parliamentary representation) yields −0.67 (W3; p = .007) and −1.10 (M3; p < .001). The within-country HDI effect is thus consistent across specifications and, if anything, strengthens under fuller control; the between-country HDI effect is also robustly negative for women (β = −0.41 to −0.56, all p ≤ .011) but near zero and non-significant for men.

## Discussion

This study investigated trends in the acceptability and prevalence of IPVAW in low- and middle-income countries over the past two decades. Consistent with recent comprehensive analyses [11,12], we confirm a widespread and enduring global decline in the social acceptability of this violence among both women and men. At the observed mean rate of decline (~1.3 percentage points per year), a country with 40% acceptability in 2000 would reach approximately 14% by 2020.

Four findings extend the existing literature. The decline is consistent across justifications, indicating a common underlying attitude rather than independently evolving context-specific beliefs. The pace of decline is correlated between women and men within countries, suggesting that normative change operates largely at the societal level. Faster HDI growth precedes faster declines in acceptability by several years, with somewhat stronger associations for HDI than for GDI; measurement caveats discussed below limit the substantive interpretation of this gap. Faster declines in acceptability also accompany faster declines in reported prevalence of physical IPVAW, providing the first cross-national link between the two trends and bearing on the puzzle of mixed progress on prevalence in earlier work [9,20].

### Development and normative change

These findings are consistent with modernization theory [13,14] and extend prior cross-sectional work [16,17] by tracking the process over time. The endogenous modernization account complements the exogenous “global cultural script” diffusion model advanced by Pierotti [11] and Swindle [15]. The two are difficult to separate in observational data: countries experiencing development improvements are also exposed to international human rights discourse. Historical examples are illustrative rather than dispositive. Most US states renounced men’s right to physically discipline their wives by the late 1870s [29], and the UK followed in 1878 [30]. These cases suggest that internal development processes can contribute to normative change alongside international diffusion, but observational data of this kind cannot adjudicate between the two pathways.

The 3–5 year lag between HDI gains and attitudinal change carries two implications. First, normative change is not immediate: it unfolds as individuals experience the effects of development (education, economic security), as egalitarian ideas diffuse through expanding schools and media, and as community standards shift in response. Second, the lag is short. If normative change operated through cohort replacement, lags would run to decades and correlations might strengthen rather than decay. The 3–5 year window is consistent with intragenerational adaptation [31] — people updating their views during their lifetimes — rather than slower demographic turnover. Recent development improvements remain the proximate correlate of current attitudes; earlier gains are not erased but are superseded.

HDI tracked attitudinal change more closely than GDI across all lags and for both genders. The GDI is constructed as a ratio of female-to-male achievement, which compresses cross-country variation and likely attenuates correlations on measurement grounds. HDI and GDI are also highly correlated in our sample (r = 0.72), so their independent contributions are difficult to disentangle. Direct measures of gender-specific institutions (legal protections, female political representation) would provide a more rigorous test. With that caveat, the somewhat stronger HDI association is consistent with general development conditions being relevant to normative change among both women and men, with men’s own education, exposure, and economic circumstances likely shaping their attitudes. The stronger HDI-acceptability correlation among men than women (r = −0.54 vs. r = −0.33) is consistent with this reading. Given these measurement caveats, our results should not be read as evidence that broad development matters more than gender-equitable development for normative change. The pattern is consistent with both processes operating jointly.

### From norms to behavior

The moderate correlation between acceptability and prevalence trends is consistent with a social-ecological understanding in which normative change is one component of a multi-level system and causal arrows may run in both directions.

Several factors can decouple the two. Strong legal frameworks can reduce violence where acceptability persists; weak enforcement can allow violence to continue where it does not. Economic stress can raise violence even as attitudes shift, while economic opportunities for women provide exit options. As violence becomes more stigmatized, survivors in some settings may become more willing to disclose it, inflating measured prevalence even as actual violence declines, while in other settings disclosure may fall. Post-conflict contexts (such as Sierra Leone in our sample) can show rising violence linked to social disruption despite declining acceptability. Despite these complexities, the positive correlation indicates that the two trends are linked at the population level.

### Policy implications

Table 2 summarizes the key findings. Two readings follow, both proportionate to the correlational design. First, the cross-national pattern is consistent with HDI-aligned investments in education (SDG 4), health (SDG 3), and economic opportunity having relevance for both human welfare and norms about violence (SDG 5), with any normative correlate of development investments emerging over a medium-term horizon rather than immediately. Existing experimental and quasi-experimental evidence shows that gender-norms interventions can reduce IPVAW [32], particularly when individual-level empowerment is combined with community-level norm change [33]; the present trends underscore the value of integrating norm-change components into economic empowerment programs, school curricula, and health-system strengthening rather than relying on standalone campaigns. Second, the population-level association between norm trends and prevalence trends supports continued investment in norm change as one component of multi-level prevention frameworks such as WHO’s RESPECT [34], alongside legal protections, accessible services for survivors, and broader development investments.

**Table 2:** Summary of key findings and policy implications.

Finding	Evidence	Policy implication
Widespread decline in IPVAW acceptability	Women: 44/47 countries (94%). Men: 33/37 countries (89%).	Norm change initiatives have broad potential for success across diverse contexts
Consistent trends across contexts	Correlations 0.50–0.83 across violence scenarios	Universal messaging about the unacceptability of beating partners is supported
Increases in human development associated with decreases in acceptability 4 years later	Women: r = -0.33; Men: r = -0.54.	Pattern is consistent with development investments being relevant to both human welfare and normative change; causal inference about violence prevention requires interventional evidence.
Trends in acceptability associated with trends in prevalence	r = 0.35 (or 0.51 if outlier is excluded)	Norm change might translate into reduced violence

### Limitations

Our design is correlational. Several factors connected to the HDI–acceptability association, including legal reforms, awareness campaigns, and women’s rights movements, may operate as mechanisms that transmit development effects, as confounders independent of development, or as both. Because we cannot empirically distinguish these roles, we do not control for these factors in the main analysis, and readers should weigh the evidence accordingly. The concerns below cover threats that operate largely independently of any specific causal pathway.

Survey research on IPVAW must consider social desirability bias [35–38]—the tendency for responses to systematically deviate from respondents’ genuine beliefs toward what is socially desirable. For our findings of declining acceptance of IPVAW, this concern shifts the interpretation from declining *private* acceptance to declining *public* acceptance: it has become less socially acceptable to endorse violence against wives. In practice, this distinction may not matter much. Public norms—what people believe they should say and what others in their community find acceptable—shape behavior through social sanctions, gossip, and community judgment, independent of private beliefs. Both private and public norms are sociologically meaningful, and both influence violence risk. Our data capture normative change in the broad sense, even if we cannot determine the precise mix of shifting private beliefs versus shifting public standards.

Organized backlash is a second concern. Anti-gender campaigns unite religious conservatives, right-wing populists, and opponents of “gender ideology” through legislation restricting reproductive rights, withdrawal from agreements such as the Istanbul Convention [39], and attacks on feminist organizations [40–42]. Our data (1999–2024) largely predate the most intensive phase of this mobilization, so the observed trends may not extrapolate to the current political landscape.

Several standard limitations apply. As a country-level analysis, we cannot identify the specific mechanisms (individual education, community norms, legal reforms) through which development affects attitudes, nor fully rule out unmeasured country-level confounders beyond those addressed in the robustness analyses. Our data are limited to low- and middle-income countries and do not include questions about infidelity, often the most widely accepted justification for violence [24], so our estimates of total acceptance are conservative. Countries with only two survey waves contribute less precise trend estimates, though such measurement error attenuates correlations toward zero rather than inflating them. Finally, our country-level averages conceal important variation within countries (urban vs. rural, rich vs. poor, different ethnic groups); future research examining these subgroups would reveal how normative change spreads through societies.

## Conclusion

The social norms that have historically justified violence against women are eroding across the globe. Beyond confirming this trend, we provide the first longitudinal evidence that the decline is associated with improvements in human development and corresponds with reductions in experienced violence. Broad-based initiatives raising living standards, health, and education are associated with normative change toward rejection of domestic violence; whether such initiatives causally contribute to reductions in violence prevalence remains to be established by designs that can support causal inference. The cross-national pattern is consistent with development conditions in which both women and men come to hold more egalitarian attitudes, though the analysis cannot establish that any specific development strategy causes this shift, nor that broad development matters more than gender-equitable development. The moderate correlation between acceptability and prevalence trends is a reminder that normative change, while a correlate of progress, is not sufficient on its own. Achieving the vision of SDG 5.2 will require comprehensive, multi-level interventions that integrate broad development investments with targeted norm-change efforts, robust legal protections, and accessible support services for survivors.

## Supporting information

S1 Appendix Fig A. Comparison of two-point and OLS trend estimates for each measure. Each point represents one country. The dashed line indicates identity; the solid line is the linear fit. The near-perfect alignment confirms that the two estimation methods produce equivalent results. Fig B. Correlation between development trends and acceptability trends across different time lags, comparing two-point and OLS estimators. Lines show Pearson correlations at lags of 1–10 years for HDI (green) and GDI (orange), using either two-point (solid) or OLS (dashed) trend estimates. Left panel: women’s acceptability. Right panel: men’s acceptability. The near-identical profiles confirm that the lag structure is robust to the trend estimation method. Fig C. Scatterplot of OLS-estimated time trends for the prevalence of IPVAW (y-axis) against OLS-estimated trends in acceptability (x-axis), in percentage points per year. Labels are ISO country codes. Compare with Fig 4, which uses two-point estimates. Fig D. Lead-lag falsification test. Each point shows the Pearson correlation between HDI change and acceptability change at different temporal offsets, shown separately for women’s acceptability (left panel) and men’s acceptability (right panel). Negative offsets (lags) indicate HDI change preceding acceptability change; positive offsets (leads) indicate HDI change following acceptability change. The asymmetry—negative lag correlations and near-zero lead correlations—supports the hypothesized temporal ordering. Table A. Sample size per country-year. Only the acceptability of IPVAW was used in countries that participated once. The sample size for the prevalence of IPVAW is smaller because only random subsamples of women are asked questions from the domestic violence module. *Note on data usage: All listed survey waves were utilized for the longitudinal analysis of the Human Development Index (HDI) and Gender Development Index (GDI) and for the associated lag analyses. For the calculation of the primary annualized rates of change in IPVAW acceptability and prevalence, only the first and last available survey waves (endpoints) for each country were used.* Table B. Hierarchical longitudinal model of acceptability of IPVAW, estimated separately for women (W1–W3) and men (M1–M3). Each row is a country-year DHS wave, inverse-variance weighted by the binomial sampling variance of the country-year proportion. The outcome and all time-varying predictors are standardised (per-sex pooled SD), so coefficients are standardised β. All specifications include country-specific random intercepts and random linear time trends, plus a fixed global linear year trend. Time-varying predictors are decomposed into country-mean (between) and deviation-from-country-mean (within) components following Bell & Jones (2015). W2/M2 adds plausibly-exogenous lagged confounders (UCDP/PRIO armed-conflict intensity; ODA as % of GNI). W3/M3 further adds partly-endogenous lagged confounders (trade openness as % of GDP; women’s parliamentary share). Cells report β [95% CI], exact p (APA). Table C. Time trends in the prevalence and acceptability of IPVAW by country. Estimates represent annual change in percentage points per year, calculated as the difference between first and last observed values divided by the number of years. 95% confidence intervals are shown in brackets. Table D. Key correlations using two-point vs. OLS trend estimates (HDI and GDI at lag = 4; acceptability vs. prevalence). The table shows that results are virtually identical regardless of trend estimation method, confirming the robustness of the main findings.
(DOCX)
