# Supplementary material for: Trends in the acceptability and prevalence of intimate partner violence: Evidence from Demographic and Health Surveys, 1999–2024
Source: PLOS Glob Public Health. 2026 Jun 24;6(6):e0006628. doi: 10.1371/journal.pgph.0006628 (PMC13293389; doi:10.1371/journal.pgph.0006628)
Supplement: S1 Appendix — Fig A. Comparison of two-point and OLS trend estimates for each measure. Each point represents one country. The dashed line indicates identity; the solid line is the linear fit. The near-perfect alignment confirms that the two estimation methods produce equivalent results. Fig B. Correlation between development trends and acceptability trends across different time lags, comparing two-point and OLS estimators. Lines show Pearson correlations at lags of 1–10 years for HDI (green) and GDI (orange), using either two-point (solid) or OLS (dashed) trend estimates. Left panel: women’s acceptability. Right panel: men’s acceptability. The near-identical profiles confirm that the lag structure is robust to the trend estimation method. Fig C. Scatterplot of OLS-estimated time trends for the prevalence of IPVAW (y-axis) against OLS-estimated trends in acceptability (x-axis), in percentage points per year. Labels are ISO country codes. Compare with Fig 4, which uses two-point estimates. Fig D. Lead-lag falsification test. Each point shows the Pearson correlation between HDI change and acceptability change at different temporal offsets, shown separately for women’s acceptability (left panel) and men’s acceptability (right panel). Negative offsets (lags) indicate HDI change preceding acceptability change; positive offsets (leads) indicate HDI change following acceptability change. The asymmetry—negative lag correlations and near-zero lead correlations—supports the hypothesized temporal ordering. Table A. Sample size per country-year. Only the acceptability of IPVAW was used in countries that participated once. The sample size for the prevalence of IPVAW is smaller because only random subsamples of women are asked questions from the domestic violence module. Note on data usage: All listed survey waves were utilized for the longitudinal analysis of the Human Development Index (HDI) and Gender Development Index (GDI) and for the associated lag analyses. For the calculation of the pri [file pgph.0006628.s001.docx]

# S1 Appendix

**
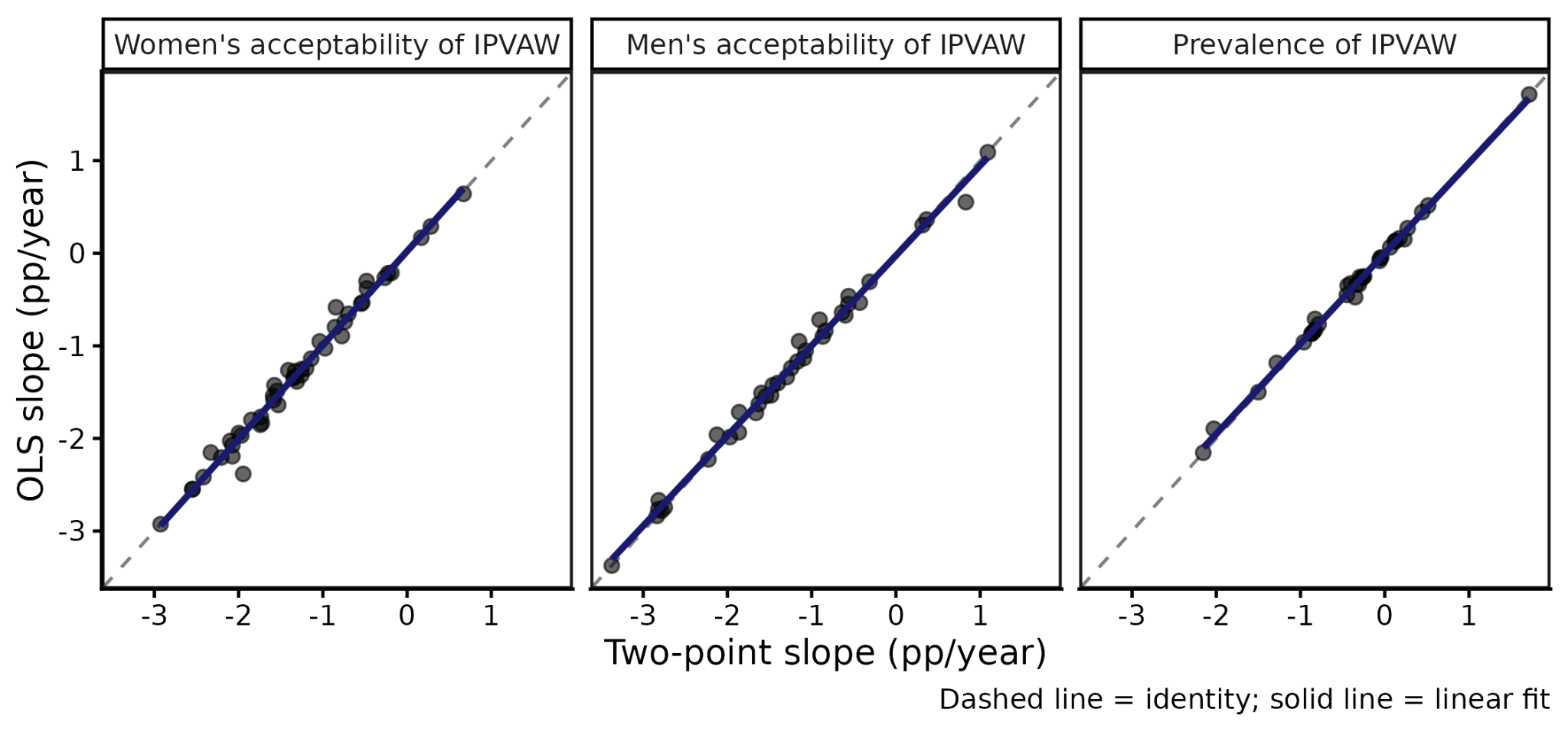
**

**Fig A**. Comparison of two-point and OLS trend estimates for each measure. Each point represents one country. The dashed line indicates identity; the solid line is the linear fit. The near-perfect alignment confirms that the two estimation methods produce equivalent results.


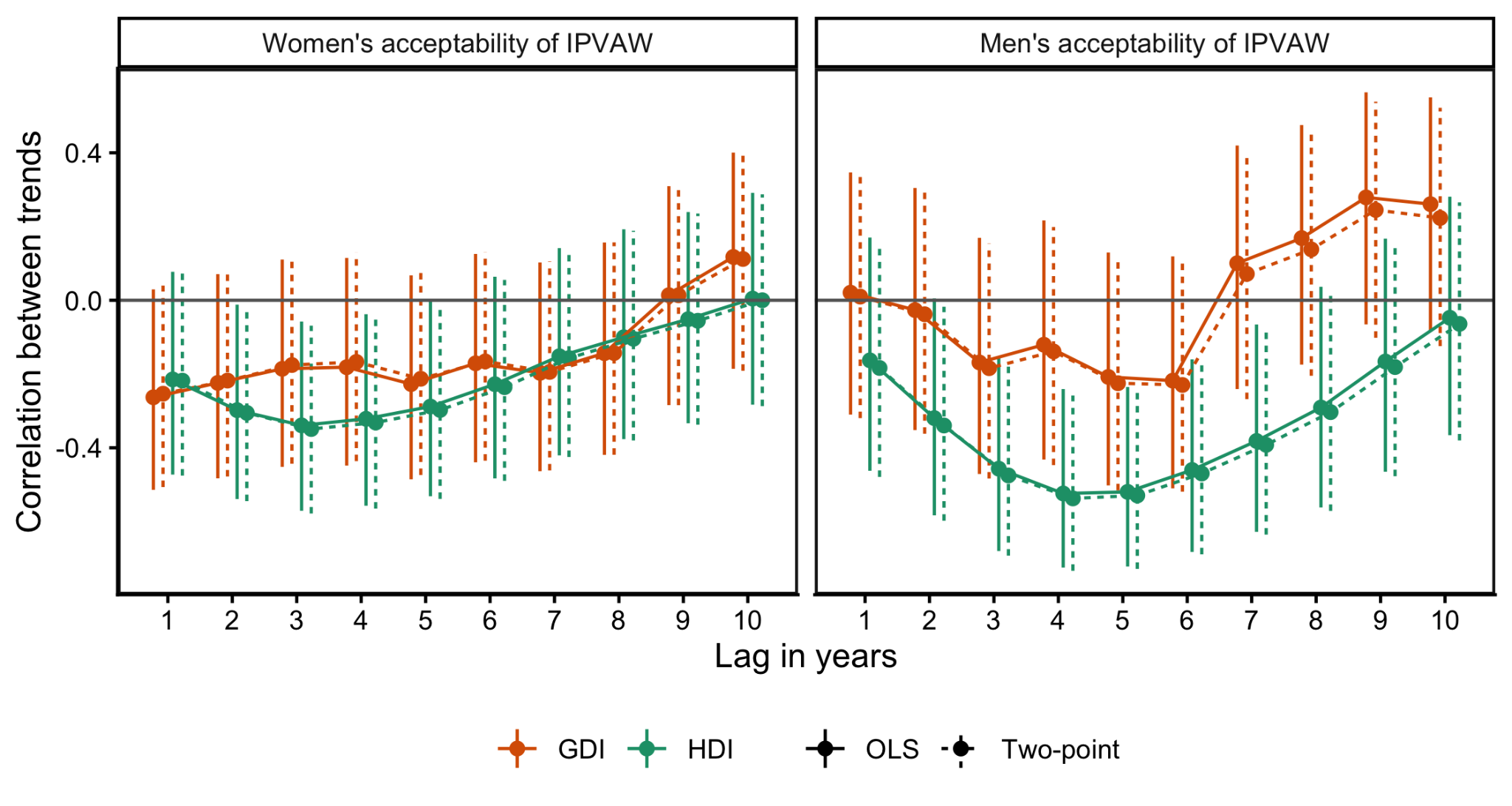
**Fig B**. Correlation between development trends and acceptability trends across different time lags, comparing two-point and OLS estimators. Lines show Pearson correlations at lags of 1–10 years for HDI (green) and GDI (orange), using either two-point (solid) or OLS (dashed) trend estimates. Left panel: women’s acceptability. Right panel: men’s acceptability. The near-identical profiles confirm that the lag structure is robust to the trend estimation method.

**
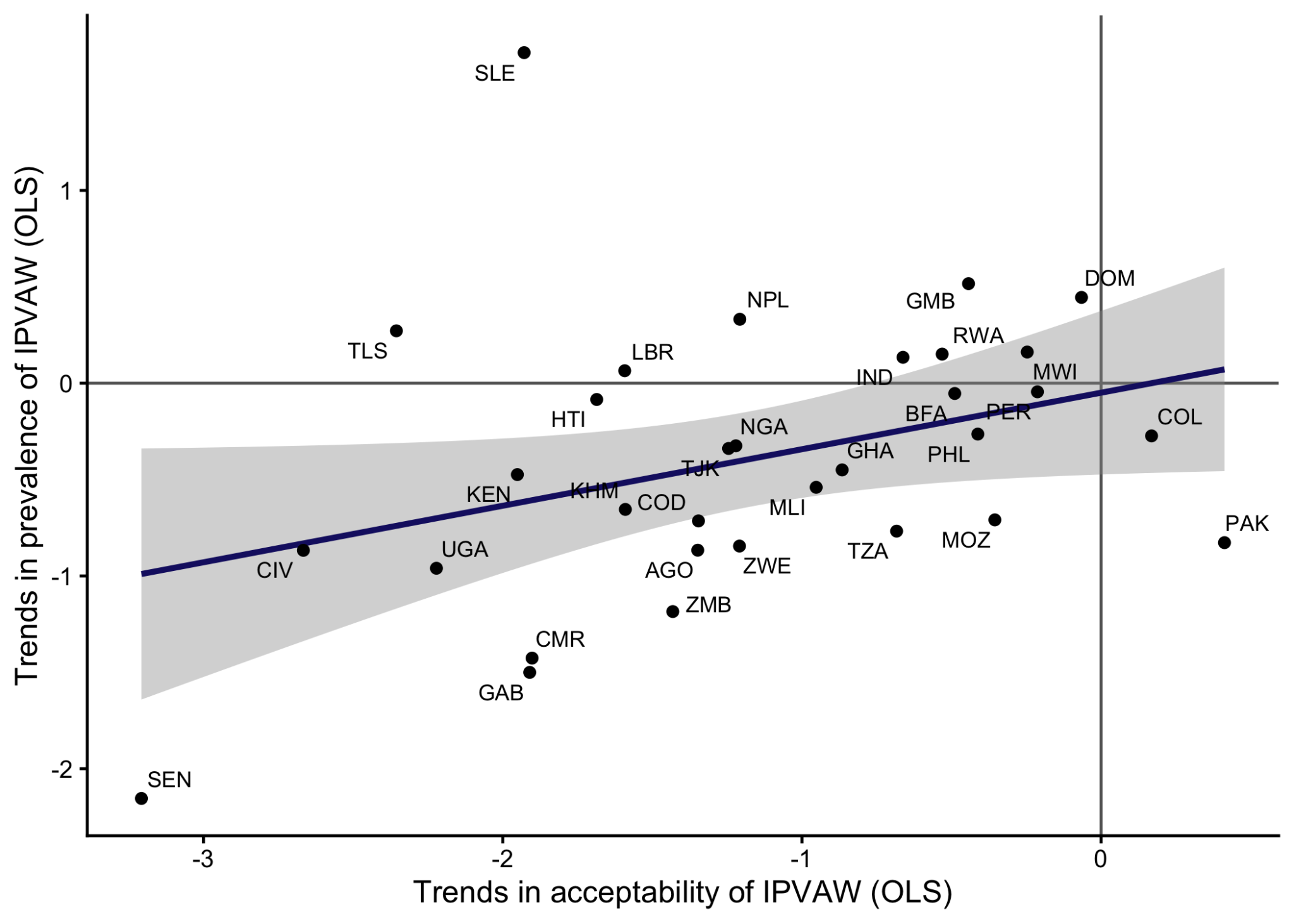
Fig C**. Scatterplot of OLS-estimated time trends for the prevalence of IPVAW (y-axis) against OLS-estimated trends in acceptability (x-axis), in percentage points per year. Labels are ISO country codes. Compare with Figure 4, which uses two-point estimates.

**
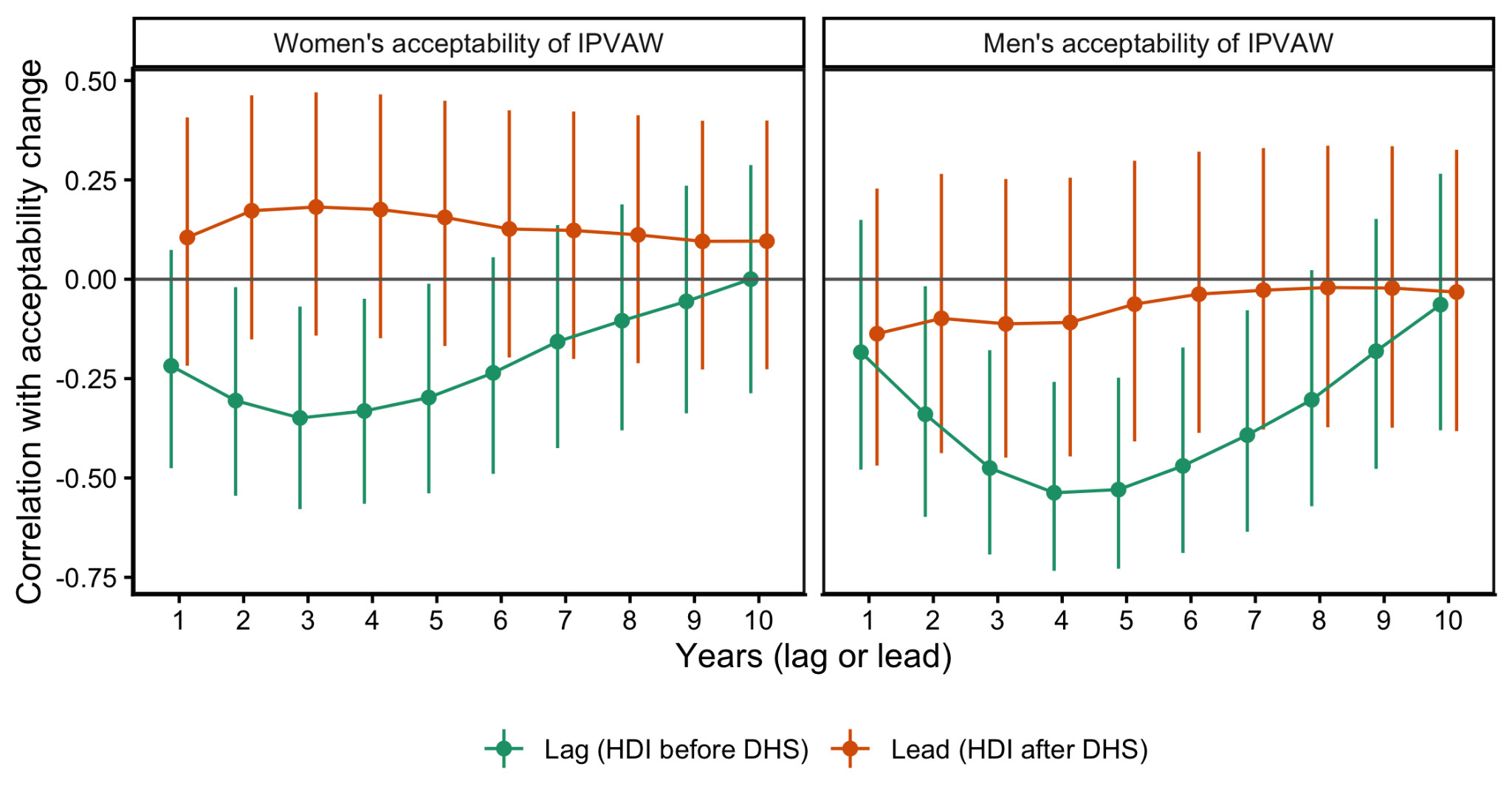
**

**Fig D.** Lead-lag falsification test. Each point shows the Pearson correlation between HDI change and acceptability change at different temporal offsets, shown separately for women's acceptability (left panel) and men's acceptability (right panel). Negative offsets (lags) indicate HDI change preceding acceptability change; positive offsets (leads) indicate HDI change following acceptability change. The asymmetry—negative lag correlations and near-zero lead correlations—supports the hypothesized temporal ordering.

**Table A**. Sample size per country-year. Only the acceptability of IPVAW was used in countries that participated once. The sample size for the prevalence of IPVAW is smaller because only random subsamples of women are asked questions from the domestic violence module. *Note on data usage: All listed survey waves were utilized for the longitudinal analysis of the Human Development Index (HDI) and Gender Development Index (GDI) and for the associated lag analyses. For the calculation of the primary annualized rates of change in IPVAW acceptability and prevalence, only the first and last available survey waves (endpoints) for each country were used.*

|  | | Women | | Men |
| --- | --- | --- | --- | --- |
| Country | Year | N acceptability | N prevalence | N acceptability |
| Afghanistan | 2015 | 29,306 | - | 10,715 |
| Albania | 2008 | 5,079 | - | 1,670 |
| Albania | 2017 | 7,582 | - | 2,227 |
| Angola | 2015 | 1,899 | 1,549 | 856 |
| Angola | 2023 | 791 | 243 | - |
| Armenia | 2000 | 4,582 | - | 1,052 |
| Armenia | 2005 | 4,450 | - | 769 |
| Armenia | 2010 | 4,015 | - | 836 |
| Armenia | 2016 | 4,309 | 3,490 | 1,408 |
| Azerbaijan | 2006 | 5,726 | 4,235 | 1,373 |
| Bangladesh | 2007 | - | 4,313 | - |
| Bangladesh | 2011 | 17,749 | - | - |
| Bangladesh | 2014 | 17,861 | - | - |
| Bangladesh | 2017 | 20,127 | - | - |
| Benin | 2001 | 3,927 | - | 1,213 |
| Benin | 2006 | 12,910 | - | 2,634 |
| Benin | 2012 | 9,775 | - | 1,935 |
| Benin | 2017 | 9,215 | 3,410 | 3,048 |
| Bolivia | 2003 | 7,876 | - | 2,043 |
| Bolivia | 2008 | 6,865 | - | - |
| Burkina Faso | 2003 | 8,836 | - | 1,524 |
| Burkina Faso | 2010 | 13,162 | 9,446 | 3,470 |
| Burkina Faso | 2021 | 9,913 | 6,625 | 3,363 |
| Burundi | 2010 | 4,098 | - | 1,513 |
| Burundi | 2016 | 7,808 | 5,092 | 2,769 |
| Cambodia | 2000 | - | 2,396 | - |
| Cambodia | 2005 | - | 2,277 | - |
| Cambodia | 2010 | 12,634 | - | 4,898 |
| Cambodia | 2014 | 12,742 | 3,450 | 3,406 |
| Cambodia | 2021 | 14,546 | 5,941 | 5,770 |
| Cameroon | 2004 | 5,998 | 1,864 | - |
| Cameroon | 2011 | 8,049 | 2,960 | 2,289 |
| Cameroon | 2018 | 6,062 | 3,320 | 1,677 |
| Chad | 2014 | 13,646 | 3,483 | 2,583 |
| Colombia | 2000 | - | 2,180 | - |
| Colombia | 2005 | - | 9,184 | - |
| Colombia | 2010 | 10,905 | 10,712 | - |
| Colombia | 2015 | 6,909 | 6,909 | 4,093 |
| Comoros | 2012 | 3,185 | 2,300 | 934 |
| Congo Democratic Republic | 2007 | 6,175 | 2,316 | - |
| Congo Democratic Republic | 2013 | 10,110 | 4,054 | 3,558 |
| Congo Democratic Republic | 2023 | 12,057 | 4,977 | 4,905 |
| Cote d'Ivoire | 2012 | 4,641 | 3,217 | 1,418 |
| Cote d'Ivoire | 2021 | 5,479 | 1,993 | 2,356 |
| Dominican Republic | 2002 | 4,469 | 1,567 | 341 |
| Dominican Republic | 2007 | 4,360 | 1,696 | 2,618 |
| Dominican Republic | 2013 | 1,489 | 1,161 | 954 |
| Egypt | 2005 | 19,412 | - | - |
| Egypt | 2008 | 16,498 | - | - |
| Egypt | 2014 | 21,760 | 6,688 | - |
| Eswatini | 2006 | 1,893 | - | 1,131 |
| Ethiopia | 2000 | 11,100 | - | 1,244 |
| Ethiopia | 2005 | 9,814 | - | 2,979 |
| Ethiopia | 2011 | 11,113 | - | 6,810 |
| Ethiopia | 2016 | 10,902 | 4,522 | 6,234 |
| Gabon | 2012 | 1,680 | 1,209 | 892 |
| Gabon | 2019 | 1,408 | 714 | 663 |
| Gambia | 2013 | 7,303 | 3,505 | 1,421 |
| Gambia | 2019 | 8,607 | 1,943 | 1,819 |
| Ghana | 2003 | 3,630 | - | 2,197 |
| Ghana | 2008 | 2,648 | 1,423 | 1,848 |
| Ghana | 2014 | 4,889 | - | 1,628 |
| Ghana | 2022 | 7,620 | 3,159 | 2,752 |
| Guatemala | 2015 | 10,203 | 3,818 | 3,514 |
| Guinea | 2005 | 6,321 | - | - |
| Guinea | 2012 | 7,036 | - | 1,650 |
| Guinea | 2018 | 7,933 | - | 1,755 |
| Guyana | 2009 | 2,016 | - | 1,232 |
| Haiti | 2000 | 6,463 | 2,459 | 1,269 |
| Haiti | 2006 | 5,517 | 2,067 | - |
| Haiti | 2012 | 7,286 | 5,514 | 3,321 |
| Haiti | 2016 | 6,540 | 3,415 | 3,158 |
| Honduras | 2005 | 5,935 | - | - |
| Honduras | 2011 | 5,916 | 4,503 | 1,630 |
| India | 2006 | 93,653 | 68,466 | 41,102 |
| India | 2015 | 91,933 | 66,013 | 63,389 |
| India | 2020 | 81,607 | 63,851 | 56,513 |
| Indonesia | 2003 | 29,455 | - | 7,324 |
| Indonesia | 2007 | 32,811 | - | 7,689 |
| Indonesia | 2012 | 34,388 | - | 8,021 |
| Indonesia | 2017 | 36,392 | - | 8,456 |
| Jordan | 2007 | - | 3,386 | - |
| Jordan | 2012 | - | 7,027 | - |
| Jordan | 2017 | - | 6,852 | - |
| Jordan | 2023 | - | 5,495 | - |
| Kenya | 2003 | 5,116 | 3,833 | 1,706 |
| Kenya | 2008 | 5,393 | 4,466 | 1,647 |
| Kenya | 2014 | 9,806 | 4,120 | 6,182 |
| Kenya | 2022 | 19,543 | 11,358 | 6,589 |
| Kyrgyz Republic | 2012 | 5,928 | 4,694 | 1,534 |
| Lesotho | 2004 | 4,636 | - | 1,077 |
| Lesotho | 2009 | 4,877 | - | 1,253 |
| Lesotho | 2014 | 4,271 | - | 1,113 |
| Lesotho | 2023 | 3,914 | 1,621 | 1,261 |
| Liberia | 2007 | 3,307 | 2,460 | 2,138 |
| Liberia | 2013 | 3,518 | - | 1,508 |
| Liberia | 2019 | 2,629 | 1,134 | 1,010 |
| Madagascar | 2004 | 4,638 | - | 1,183 |
| Madagascar | 2008 | 11,641 | - | 4,287 |
| Madagascar | 2021 | 10,887 | 4,615 | 4,248 |
| Malawi | 2000 | 10,590 | - | 1,746 |
| Malawi | 2004 | 9,166 | 7,724 | 1,993 |
| Malawi | 2010 | 16,221 | 4,690 | 3,491 |
| Malawi | 2015 | 17,846 | 4,996 | 3,933 |
| Malawi | 2024 | 14,665 | 12,355 | 4,473 |
| Maldives | 2009 | 7,101 | - | 1,356 |
| Maldives | 2016 | - | 3,343 | - |
| Mali | 2001 | 10,826 | - | 1,653 |
| Mali | 2006 | 11,839 | 8,422 | - |
| Mali | 2012 | 8,703 | 3,052 | 2,366 |
| Mali | 2018 | 8,545 | 3,314 | 2,425 |
| Mali | 2023 | 13,281 | 3,418 | 3,469 |
| Mauritania | 2020 | 11,477 | 3,307 | 2,255 |
| Moldova | 2005 | 4,989 | 3,242 | 1,192 |
| Morocco | 2003 | 9,710 | - | - |
| Mozambique | 2003 | 1,975 | - | 581 |
| Mozambique | 2011 | 5,985 | 3,364 | 1,400 |
| Mozambique | 2015 | 2,382 | 1,219 | 1,192 |
| Mozambique | 2022 | 3,592 | 1,450 | 1,498 |
| Myanmar | 2016 | 8,739 | 3,425 | 3,042 |
| Namibia | 2006 | 2,395 | - | 739 |
| Namibia | 2013 | 2,048 | 764 | 704 |
| Nepal | 2001 | 8,723 | - | 1,862 |
| Nepal | 2006 | 8,638 | - | 2,632 |
| Nepal | 2011 | - | 3,504 | - |
| Nepal | 2016 | 10,213 | 3,818 | 2,719 |
| Nepal | 2022 | 11,683 | 4,359 | 3,223 |
| Nicaragua | 2001 | 4,322 | - | - |
| Niger | 2006 | 7,804 | - | - |
| Niger | 2012 | 9,896 | - | 2,160 |
| Nigeria | 2003 | 5,318 | - | 936 |
| Nigeria | 2008 | 24,616 | 18,590 | 7,098 |
| Nigeria | 2013 | 28,040 | 21,337 | 8,506 |
| Nigeria | 2018 | 29,862 | 8,463 | 6,499 |
| Nigeria | 2024 | 25,956 | 20,085 | 5,751 |
| Pakistan | 2012 | 13,527 | 3,669 | 3,125 |
| Pakistan | 2017 | 15,065 | 4,080 | 3,675 |
| Papua New Guinea | 2017 | 8,969 | 3,202 | 3,480 |
| Peru | 2004 | 10,490 | 9,960 | - |
| Peru | 2007 | 10,490 | 9,960 | - |
| Peru | 2009 | 6,807 | 5,535 | - |
| Peru | 2010 | 6,281 | 4,986 | - |
| Peru | 2011 | 5,997 | 4,866 | - |
| Peru | 2012 | 6,033 | 4,837 | - |
| Philippines | 2003 | 8,096 | - | 2,180 |
| Philippines | 2008 | 7,466 | 5,846 | - |
| Philippines | 2013 | 8,044 | 6,203 | - |
| Philippines | 2017 | 12,037 | 9,692 | - |
| Philippines | 2022 | 11,213 | 8,852 | - |
| Rwanda | 2000 | 4,052 | - | 817 |
| Rwanda | 2005 | 3,856 | 1,355 | 1,393 |
| Rwanda | 2010 | 5,257 | 2,156 | 1,963 |
| Rwanda | 2015 | 5,040 | 1,125 | 1,839 |
| Rwanda | 2019 | 5,085 | 1,102 | 1,810 |
| Sao Tome and Principe | 2008 | 125 | 105 | 76 |
| Senegal | 2005 | 10,286 | - | - |
| Senegal | 2010 | 11,225 | - | 1,701 |
| Senegal | 2012 | 5,996 | - | - |
| Senegal | 2014 | 6,030 | - | 1,199 |
| Senegal | 2015 | 6,312 | - | 1,413 |
| Senegal | 2016 | 6,390 | - | 1,237 |
| Senegal | 2017 | 12,009 | 2,631 | 2,473 |
| Senegal | 2018 | 6,754 | 1,491 | 1,375 |
| Senegal | 2019 | 6,251 | 1,463 | 1,243 |
| Sierra Leone | 2008 | 5,039 | - | 1,626 |
| Sierra Leone | 2013 | 11,159 | 4,106 | 3,463 |
| Sierra Leone | 2019 | 9,919 | 3,798 | 3,186 |
| South Africa | 2016 | 2,164 | - | 598 |
| Tajikistan | 2012 | 6,836 | 4,343 | - |
| Tajikistan | 2017 | 8,064 | 5,221 | - |
| Tajikistan | 2023 | 7,685 | 5,440 | - |
| Tanzania | 2004 | 6,784 | - | 1,341 |
| Tanzania | 2010 | 6,688 | 5,123 | 1,258 |
| Tanzania | 2015 | 7,109 | 5,450 | 1,286 |
| Tanzania | 2022 | 7,904 | 3,171 | 2,750 |
| Timor-Leste | 2009 | 7,930 | 2,017 | 2,029 |
| Timor-Leste | 2016 | 6,887 | 3,208 | 1,794 |
| Togo | 2013 | 5,477 | 4,122 | 1,737 |
| Turkey | 2013 | 7,144 | - | - |
| Uganda | 2000 | 3,736 | - | 1,063 |
| Uganda | 2006 | 4,951 | 1,294 | 1,281 |
| Uganda | 2011 | 3,840 | 958 | 947 |
| Uganda | 2016 | 6,769 | 3,695 | 1,939 |
| Ukraine | 2007 | 4,818 | 2,180 | 1,905 |
| Yemen | 2013 | 16,578 | - | - |
| Zambia | 2002 | 5,700 | - | 1,156 |
| Zambia | 2007 | 5,082 | 4,105 | 3,374 |
| Zambia | 2013 | 11,462 | 9,223 | 7,484 |
| Zambia | 2018 | 9,190 | 7,242 | 5,838 |
| Zambia | 2024 | 9,061 | 7,084 | 5,867 |
| Zimbabwe | 1999 | 3,652 | - | - |
| Zimbabwe | 2005 | 6,163 | 4,623 | 3,157 |
| Zimbabwe | 2010 | 6,423 | 4,971 | 3,657 |
| Zimbabwe | 2015 | 6,856 | 5,462 | 4,246 |

**Table B.** Hierarchical longitudinal model of acceptability of IPVAW, estimated separately for women (W1–W3) and men (M1–M3). Each row is a country-year DHS wave, inverse-variance weighted by the binomial sampling variance of the country-year proportion. The outcome and all time-varying predictors are standardised (per-sex pooled SD), so coefficients are standardised β. All specifications include country-specific random intercepts and random linear time trends, plus a fixed global linear year trend. Time-varying predictors are decomposed into country-mean (between) and deviation-from-country-mean (within) components following Bell & Jones (2015). W2/M2 adds plausibly-exogenous lagged confounders (UCDP/PRIO armed-conflict intensity; ODA as % of GNI). W3/M3 further adds partly-endogenous lagged confounders (trade openness as % of GDP; women’s parliamentary share). Cells report β [95% CI], exact p (APA).

|  | W1 | W2 | W3 | M1 | M2 | M3 |
| --- | --- | --- | --- | --- | --- | --- |
| (Intercept) | 0.055 [-0.188, 0.298], *p* = .651 | 0.044 [-0.201, 0.289], *p* = .720 | 0.055 [-0.215, 0.324], *p* = .684 | 0.035 [-0.289, 0.359], *p* = .828 | 0.043 [-0.291, 0.377], *p* = .795 | 0.062 [-0.295, 0.419], *p* = .724 |
| ***Within-country (deviation from country mean)*** | | | | | | |
| HDI (within-country) | -0.493 [-0.884, -0.102], *p* = .014 | -0.558 [-0.955, -0.161], *p* = .006 | -0.672 [-1.161, -0.184], *p* = .007 | -1.042 [-1.507, -0.577], *p* < .001 | -0.913 [-1.425, -0.401], *p* < .001 | -1.103 [-1.718, -0.488], *p* < .001 |
| Armed conflict (within) | — | -0.071 [-0.145, 0.004], *p* = .063 | -0.068 [-0.144, 0.008], *p* = .080 | — | 0.014 [-0.099, 0.128], *p* = .801 | 0.012 [-0.130, 0.155], *p* = .864 |
| ODA % GNI (within) | — | -0.059 [-0.158, 0.039], *p* = .236 | -0.142 [-0.314, 0.031], *p* = .106 | — | 0.131 [-0.036, 0.299], *p* = .122 | 0.195 [-0.013, 0.403], *p* = .066 |
| Trade openness (within) | — | — | 0.026 [-0.081, 0.133], *p* = .629 | — | — | -0.122 [-0.286, 0.042], *p* = .143 |
| Women in parliament (within) | — | — | -0.001 [-0.140, 0.139], *p* = .991 | — | — | 0.104 [-0.141, 0.349], *p* = .397 |
| ***Between-country (country-mean level)*** | | | | | | |
| HDI (between-country) | -0.564 [-0.803, -0.325], *p* < .001 | -0.447 [-0.738, -0.157], *p* = .003 | -0.408 [-0.717, -0.099], *p* = .011 | -0.077 [-0.352, 0.199], *p* = .577 | -0.033 [-0.375, 0.309], *p* = .846 | 0.050 [-0.326, 0.426], *p* = .788 |
| Armed conflict (between) | — | 0.126 [-0.166, 0.418], *p* = .387 | 0.228 [-0.114, 0.570], *p* = .185 | — | 0.253 [-0.074, 0.581], *p* = .124 | 0.550 [0.154, 0.946], *p* = .009 |
| ODA % GNI (between) | — | 0.193 [-0.134, 0.520], *p* = .242 | 0.283 [-0.163, 0.729], *p* = .207 | — | 0.018 [-0.332, 0.367], *p* = .919 | 0.434 [-0.146, 1.014], *p* = .137 |
| Trade openness (between) | — | — | 0.035 [-0.224, 0.295], *p* = .784 | — | — | 0.185 [-0.117, 0.487], *p* = .218 |
| Women in parliament (between) | — | — | -0.014 [-0.322, 0.294], *p* = .927 | — | — | -0.066 [-0.432, 0.300], *p* = .714 |
| ***Global time trend*** | | | | | | |
| Year (per decade, global trend) | -0.180 [-0.440, 0.080], *p* = .172 | -0.154 [-0.410, 0.102], *p* = .237 | -0.101 [-0.406, 0.204], *p* = .512 | 0.081 [-0.287, 0.449], *p* = .664 | 0.044 [-0.336, 0.424], *p* = .818 | 0.242 [-0.216, 0.701], *p* = .295 |
| ***Random effects (SD, correlation)*** | | | | | | |
| σ intercept (country) | 0.790 | 0.794 | 0.831 | 0.904 | 0.931 | 0.892 |
| σ slope of year (country) | 0.271 | 0.253 | 0.277 | 0.381 | 0.378 | 0.354 |
| Cor (intercept, slope) | -0.302 | -0.390 | -0.570 | -0.717 | -0.769 | -0.801 |
| σ residual | 0.207 | 0.205 | 0.184 | 0.257 | 0.255 | 0.260 |
| ***Goodness-of-fit*** | | | | | | |
| Countries | 47 | 47 | 44 | 37 | 37 | 34 |
| Observations | 160 | 158 | 130 | 122 | 120 | 96 |
| Marginal R² | 0.341 | 0.351 | 0.324 | 0.185 | 0.202 | 0.253 |
| Conditional R² | 0.958 | 0.959 | 0.967 | 0.936 | 0.941 | 0.933 |
| AIC | 233.1 | 243.7 | 215.6 | 234.5 | 245.2 | 213.8 |
| BIC | 257.7 | 280.4 | 261.4 | 257.0 | 278.7 | 254.8 |

**Table C**. Time trends in the prevalence and acceptability of IPVAW by country. Estimates represent annual change in percentage points per year, calculated as the difference between first and last observed values divided by the number of years. 95% confidence intervals are shown in brackets.

| Country | Acceptability of IPVAW among women | Prevalence of IPVAW | Acceptability of IPVAW among men |
| --- | --- | --- | --- |
| Albania | -2.93 [-3.09, -2.76] | - | -2.74 [-3.06, -2.42] |
| Angola | -1.35 [-1.74, -0.96] | -0.87 [-1.49, -0.24] | — |
| Armenia | -1.53 [-1.64, -1.42] | - | -1.66 [-1.90, -1.43] |
| Bangladesh | -2.07 [-2.22, -1.93] | - | - |
| Benin | -1.95 [-2.06, -1.83] | - | -1.15 [-1.33, -0.97] |
| Bolivia | -0.53 [-0.79, -0.28] | - | - |
| Burkina Faso | -1.41 [-1.48, -1.33] | -0.05 [-0.14, 0.03] | -1.24 [-1.40, -1.09] |
| Burundi | -2.21 [-2.49, -1.92] | - | -1.54 [-2.04, -1.05] |
| Cambodia | -0.77 [-0.88, -0.67] | -0.44 [-0.51, -0.37] | -0.43 [-0.57, -0.29] |
| Cameroon | -1.97 [-2.09, -1.85] | -0.26 [-0.42, -0.10] | -1.63 [-2.04, -1.22] |
| Colombia | 0.17 [0.08, 0.26] | -0.34 [-0.44, -0.24] | - |
| Congo Democratic Republic | -1.25 [-1.34, -1.17] | -2.03 [-2.18, -1.88] | -0.84 [-1.05, -0.62] |
| Cote d'Ivoire | -2.55 [-2.76, -2.34] | -0.87 [-1.09, -0.64] | -2.78 [-3.11, -2.45] |
| Dominican Republic | -0.22 [-0.31, -0.14] | 0.44 [0.10, 0.79] | -0.31 [-0.56, -0.07] |
| Egypt | -1.57 [-1.68, -1.47] | - | - |
| Ethiopia | -1.31 [-1.38, -1.24] | - | -2.82 [-2.98, -2.65] |
| Gabon | -2.42 [-2.87, -1.97] | -1.50 [-1.95, -1.05] | -1.40 [-2.06, -0.74] |
| Gambia | -1.25 [-1.51, -0.99] | 0.52 [0.27, 0.76] | 0.36 [-0.15, 0.88] |
| Ghana | -1.59 [-1.69, -1.49] | -0.45 [-0.61, -0.29] | -0.87 [-0.99, -0.75] |
| Guinea | -1.33 [-1.43, -1.23] | - | -2.23 [-2.76, -1.69] |
| Haiti | -1.72 [-1.82, -1.63] | -0.06 [-0.17, 0.05] | -1.48 [-1.65, -1.31] |
| Honduras | -0.54 [-0.75, -0.33] | - | - |
| India | -0.70 [-0.73, -0.66] | 0.13 [0.10, 0.16] | -0.60 [-0.65, -0.56] |
| Indonesia | 0.28 [0.23, 0.33] | - | 0.32 [0.23, 0.40] |
| Jordan | - | -0.30 [-0.38, -0.21] | - |
| Kenya | -2.00 [-2.07, -1.92] | -0.35 [-0.44, -0.27] | -2.12 [-2.25, -2.00] |
| Lesotho | -1.54 [-1.64, -1.44] | - | -1.30 [-1.50, -1.09] |
| Liberia | -2.55 [-2.75, -2.34] | 0.06 [-0.22, 0.35] | -0.64 [-0.89, -0.39] |
| Madagascar | 0.67 [0.58, 0.76] | - | 0.83 [0.70, 0.96] |
| Malawi | -0.74 [-0.79, -0.69] | 0.17 [0.12, 0.22] | -0.56 [-0.65, -0.47] |
| Mali | -0.84 [-0.89, -0.80] | -0.24 [-0.33, -0.16] | -1.07 [-1.20, -0.94] |
| Mozambique | -2.07 [-2.20, -1.95] | -0.83 [-1.03, -0.62] | -1.46 [-1.69, -1.23] |
| Namibia | -1.14 [-1.54, -0.73] | - | -2.84 [-3.46, -2.21] |
| Nepal | -0.48 [-0.54, -0.42] | 0.12 [-0.01, 0.25] | -0.91 [-1.03, -0.79] |
| Niger | -1.59 [-1.81, -1.36] | - | - |
| Nigeria | -2.10 [-2.16, -2.04] | -0.40 [-0.44, -0.36] | -1.60 [-1.75, -1.44] |
| Pakistan | -0.27 [-0.51, -0.02] | -0.83 [-1.17, -0.49] | 1.09 [0.64, 1.55] |
| Peru | -0.19 [-0.27, -0.10] | -0.04 [-0.17, 0.08] | - |
| Philippines | -0.86 [-0.92, -0.80] | -0.27 [-0.32, -0.21] | - |
| Rwanda | -1.04 [-1.14, -0.93] | 0.23 [0.02, 0.45] | -1.86 [-2.06, -1.66] |
| Senegal | -1.85 [-1.96, -1.74] | -2.15 [-2.96, -1.35] | -0.57 [-0.88, -0.25] |
| Sierra Leone | -1.74 [-1.88, -1.59] | 1.71 [1.36, 2.07] | -2.82 [-3.08, -2.56] |
| Tajikistan | -1.20 [-1.34, -1.06] | -0.31 [-0.43, -0.18] | - |
| Tanzania | -0.48 [-0.57, -0.39] | -0.79 [-0.96, -0.61] | -1.09 [-1.27, -0.92] |
| Timor-Leste | -1.34 [-1.50, -1.17] | 0.27 [-0.11, 0.66] | -3.37 [-3.76, -2.99] |
| Uganda | -1.74 [-1.85, -1.63] | -0.96 [-1.26, -0.66] | -1.97 [-2.19, -1.75] |
| Zambia | -2.33 [-2.39, -2.27] | -1.28 [-1.39, -1.18] | -1.86 [-2.00, -1.73] |
| Zimbabwe | -0.97 [-1.09, -0.85] | -0.84 [-1.00, -0.69] | -1.17 [-1.38, -0.96] |

**Table D**. Key correlations using two-point vs. OLS trend estimates (HDI and GDI at lag = 4; acceptability vs. prevalence). The table shows that results are virtually identical regardless of trend estimation method, confirming the robustness of the main findings.

| Analysis | Two-point r [95% CI] | OLS r [95% CI] |
| --- | --- | --- |
| HDI vs. women's acceptability of IPVAW | -0.33 [-0.56, -0.05] | -0.32 [-0.56, -0.04] |
| HDI vs. men's acceptability of IPVAW | -0.54 [-0.73, -0.26] | -0.52 [-0.72, -0.24] |
| GDI vs. women's acceptability of IPVAW | -0.17 [-0.44, 0.13] | -0.18 [-0.45, 0.11] |
| GDI vs. men's acceptability of IPVAW | -0.14 [-0.45, 0.20] | -0.12 [-0.43, 0.22] |
| Acceptability vs. prevalence | 0.35 [-0.00, 0.62] | 0.35 [-0.00, 0.62] |
