# Supplementary material for: Trends in the acceptability and prevalence of intimate partner violence: Evidence from Demographic and Health Surveys, 1999–2024
Source: PLOS Glob Public Health. 2026 Jun 24;6(6):e0006628. doi: 10.1371/journal.pgph.0006628 (PMC13293389; doi:10.1371/journal.pgph.0006628)
Supplement: Attachment — Submitted filename: renamed_2a1d4.docx [file pgph.0006628.s003.docx]

Response to Reviewers — PGPH-D-26-00404

*Irina Vartanova, Kimmo Eriksson, Pontus Strimling*

We thank the Editor and reviewers for their careful engagement with our manuscript. Their feedback prompted substantial revisions to the theoretical framing, analytic presentation, and interpretive language. Below we describe the specific changes made in this revision, with verbatim quotations from the revised manuscript where relevant.

We have also taken this opportunity to incorporate four newly released DHS surveys (Angola 2023–24, Malawi 2024, Nigeria 2024, Zambia 2024), bringing the total to 47 countries with women’s acceptability trends; substantive conclusions are unchanged.

## Response to Editor

## Editor Concern 1: “*strengthen and rebalance the theoretical framing, including engagement with alternative explanatory perspectives”*

Response: We substantially expanded the theoretical framing beyond modernization theory and now state explicitly that the design cannot adjudicate among endogenous and exogenous explanations for normative change. See R1.1 and R1.2.

## Editor Concern 2: “*revise the manuscript to ensure that causal language does not exceed what can be supported by the design”*

Response: We revised causal language throughout so the manuscript now frames the findings as associations and explicitly avoids directional causal claims. See R1.5 and R1.7.

## Editor Concern 3: “*strengthen or more transparently justify the analytic strategy, including robustness analyses using all available survey waves”*

Response: We now justify the two-point estimator in the Methods and added a Results subsection, "Robustness Analyses," reporting OLS trend estimates, a lead-lag falsification test, and a hierarchical longitudinal model. See R1.4 and R1.5.

## Editor Concern 4: “*expand the discussion of measurement limitations, including the construction and cross-context validity of the attitudinal measure”*

Response: We expanded the measurement discussion in the Measures, Results, and Limitations sections, including the binary composite construction, the omission of infidelity-related justifications, and the question of cross-context validity. See R1.3.

## Editor Concern 5: “*address social desirability and reporting bias more fully”*

Response: We expanded the Limitations section to address social desirability and reporting bias directly, grounding the issue in experimental evidence and clarifying why reporting bias alone is unlikely to explain the full pattern. See R1.8.

## Editor Concern 6: “*interpret the HDI versus GDI findings more cautiously”*

Response: The revised Discussion subsection "Development and normative change" now discusses GDI measurement properties, reports the HDI-GDI correlation, and treats the HDI-GDI contrast more cautiously. See R1.6.

## Editor Concern 7: “*moderate the interpretation of the association between attitudes and prevalence”*

Response: We moderated the interpretation of the acceptability-prevalence association in the Abstract, Discussion, Table 2, and Conclusion, and now explicitly discuss bidirectional causation and reporting dynamics. See R1.7.

## Editor Concern 8: “*revise the manuscript for clarity, concision, consistency of terminology, table presentation, and standard English throughout”*

Response: We revised the manuscript throughout for clarity, concision, and standard English:

Clarity: Key concepts and abbreviations are now defined at first use.

Concision: The Discussion was tightened by reducing restatement of results; findings are referenced concisely rather than re-described. We removed interpretive statements from the Results section (see R2.15, R2.17, R2.18).

Consistency of terminology: "IPVAW" is used consistently throughout, with all acronyms expanded at first use and redundant redefinitions removed. The manuscript uses "acceptability of IPVAW" consistently, following Reviewer 2's suggestion (R2.14) that "acceptability" is clearer than "acceptance" for readers unfamiliar with the Methods.

Standard English: The Methods section uses consistent active voice ("We compiled," "We calculated," "We measured"). We replaced informal phrasing throughout (e.g., "we don't know which lag to expect" became "the lag between development improvements and normative change is theoretically uncertain").

We moved Figure 1 to the Methods section (before Statistical Analysis) following Reviewer 2's suggestion.

## Response to Reviewer 1

## R1.1: Theoretical framing relies heavily on modernization theory

*In my opinion, the most fundamental limitation concerns theoretical framing. The authors rely heavily on modernization theory to interpret the observed associations between development and normative change. However, I find the engagement with the theory to be limited and insufficiently critical. The colleagues, on the one hand, present modernization as the primary explanatory framework, while, on the other hand, they only briefly mention and without substantive engagement, alternative perspectives, - such as global cultural diffusion, institutional diffusion through international organizations, transnational feminist advocacy networks, or the theory of the world polity. As a result, their theoretical discussion seems somewhat confirmatory rather than truly explanatory. Their analysis is structured in a way that implicitly assumes development-driven value change rather than rigorously testing competing explanations for the observed trends.*

Response: The revised Background now engages with four complementary theoretical frameworks rather than relying on modernization theory alone. Where the original submission mentioned alternative perspectives only briefly, the revision develops each with specific evidence and theoretical grounding: Pierotti’s (2013) demonstration that structural changes could not explain declining IPVAW acceptability, pointing instead to global cultural script diffusion; Swindle’s (2023) identification of mass media as the primary diffusion pathway; True & Mintrom’s (2001) evidence that transnational networks drove gender mainstreaming across 157 countries independently of domestic development; and Htun & Weldon’s (2012) finding that autonomous feminist mobilization was the strongest predictor of progressive VAW policy across 70 countries over four decades.

The revised Background paragraph states:

“Other scholars have emphasized exogenous explanations for normative change that operate independently of domestic economic development. Pierotti, analyzing DHS data from 26 countries, found that structural changes within countries—rising urbanization, education, and media access—could not account for the decline in IPVAW acceptability; she attributed the trend to the diffusion of a global cultural script condemning domestic violence, transmitted through international organizations, media, and education systems [11]. Swindle extended this by showing that mass media exposure is a primary conduit for normative diffusion, with media access predicting individual-level attitude change [15]. A parallel pathway operates through institutional mechanisms: True and Mintrom demonstrated that gender mainstreaming adoption across 157 countries was driven by transnational networks and participation in UN women’s conferences, not by domestic development levels [18]. Htun and Weldon, analyzing 70 countries over four decades, found that autonomous feminist mobilization—not national wealth or women’s political representation—was the strongest predictor of progressive policy on violence against women [19].”

The Discussion reinforces this:

"The endogenous modernization account complements the exogenous “global cultural script” diffusion model advanced by Pierotti [11] and Swindle [15]. The two are difficult to separate in observational data: countries experiencing development improvements are also exposed to international human rights discourse."

## R1.2: Empirical strategy cannot distinguish endogenous from exogenous processes

*I wish to emphasize that this issue is compounded by the fact that the empirical strategy cannot convincingly distinguish between endogenous development processes and exogenous norm diffusion. In my view, both processes could plausibly produce the same empirical pattern: countries experiencing improvements in development indicators might simultaneously be more exposed to global human rights discourses, international development programs, and media narratives condemning domestic violence. I would like to draw the attention of colleagues to the fact that without incorporating variables capturing global institutional diffusion or policy adoption, the analysis risks attributing causal explanatory power to development indicators that may simply correlate with broader processes of globalization and norm institutionalization.*

Response: We agree that the design cannot cleanly distinguish endogenous development from exogenous norm diffusion, and we now say so explicitly in the Background:

“These endogenous and exogenous mechanisms are not mutually exclusive; countries experiencing development improvements may simultaneously face greater exposure to international human rights discourses. Development may facilitate normative diffusion by expanding education, media access, and civic capacity—making it difficult to disentangle whether norm change reflects endogenous value shifts or increased reception of exogenous global scripts. Our analysis documents associations between development trends and normative change but cannot adjudicate between these complementary explanations.”

To show that the association is not limited to a simple bivariate cross-country correlation, we added a lead-lag falsification test and a multilevel longitudinal model with time-varying controls (S4 Fig and S4 Table); full results are reported under R1.5.

The Limitations section now also states:

"Unmeasured confounders such as conflict dynamics and shifts in reporting behavior could drive both development and normative change, though the Results robustness analyses constrain the most obvious alternatives."

## R1.3: Binary composite indicator is conceptually blunt

*A second major concern relates to the measurement of the key attitudinal variable. If I understood correctly, the operationalization of "acceptance of intimate partner violence" relies on a composite indicator derived from five DHS questions asking whether a husband is justified in hitting or beating his wife in specific circumstances (e.g., burning food, arguing, going out without informing him). Respondents are coded as accepting violence if they endorse any of these scenarios. Here of course, although this operationalization follows established practice in DHS-based research, I find that it is conceptually blunt. It conflates very different normative positions into a single binary measure and does not capture gradations in moral justification or contextual reasoning. Furthermore, as the authors acknowledge, the DHS questions omit infidelity-related justifications, which are widely documented as among the most accepted triggers for partner violence in many societies. Consequently, do not colleagues believe that the indicator likely underestimates overall normative acceptance and may also vary in validity across cultural contexts?*

Response: We acknowledge that the binary composite is a simplification that does not capture gradations in moral justification. However, binary coding is standard practice in DHS-based research on IPVAW acceptability (e.g., Bergenfeld et al. 2025; Pierotti 2013) and is appropriate for our purpose: estimating population-level trends over time. The binary measure captures whether a respondent endorses any justification for violence, and the strong intercorrelations across the five contexts (r = 0.50-0.83) demonstrate that these items tap a common underlying attitude rather than distinct context-specific beliefs. For trend analysis, this composite summarizes normative stance.

On the infidelity omission: the Measures section now explicitly notes that "the DHS does not include questions about justifying violence in response to suspected or actual infidelity. Research using the WHO Multi-country Study methodology has found that infidelity is consistently the most widely accepted justification for violence in many contexts [24]. Thus, our DHS-based estimates likely represent a conservative (lower-bound) measure of total IPVAW acceptability." This means our trends may understate the true level of acceptability, but the direction and relative magnitude of trends should be unaffected, since there is no reason to expect the omission to differentially bias change rates across countries.

## R1.4: Using only first and last survey observations ignores intermediate data

*The methodological strategy used to estimate temporal trends is also problematic. If again I have understood correctly, the authors calculate country-specific trends using only the first and last available survey observations, dividing the difference by the time interval between them. But, while this approach produces a simple measure of average change, it ignores all intermediate data points and assumes linear change over time. In my opinion, in countries with multiple DHS waves, this decision results in a substantial loss of information and prevents the detection of non-linear dynamics, reversals, or acceleration in normative change. The authors briefly state that results are "virtually identical" when using OLS estimates based on all available time points, but these alternative analyses are not presented in sufficient detail to allow readers to assess their robustness.*

Response: We agree that relying only on the first and last observations could miss important dynamics, so we added a robustness section using all available waves. The Results now state:

"As a robustness check, we re-estimated all country-level trends using ordinary least squares (OLS) regression fitted to all available survey waves, rather than the two-point estimates used in the main analysis. OLS slopes were highly correlated with the two-point estimates across all measures (S1 Fig), and re-running the key analyses with OLS-derived trends produced virtually identical lag profiles (S2 Fig), acceptability–prevalence associations (S3 Fig), and summary statistics (S3 Table). This consistency indicates that our findings are not sensitive to the choice of trend estimation method."

We agree that non-linear dynamics are important to consider. For the 21 countries included in Pierotti's original study — the subset with the longest available time series — we compared rates of decline across two sub-periods. Women's acceptability of IPVAW fell at 1.82 percentage points per year in 1999–2010 and at 0.91 percentage points per year in 2011–2024, a clear deceleration. The revised manuscript now highlights this dynamic directly.

## R1.5: Core analyses rely on simple Pearson correlations without controlling for confounders

*Although I am not a statistician and in general speaking, I find that the statistical design remains relatively weak for the type of causal interpretation suggested in the discussion. The core analyses rely on simple Pearson correlations between country-level trend estimates. While the authors introduce temporal lags between development indicators and attitudinal change, the analysis remains essentially correlational and does not control for potential confounders. I invite colleagues to reflect on how many factors (including legal reforms, public awareness campaigns, expansion of women's rights movements, conflict dynamics, or shifts in reporting behavior), could simultaneously influence both development indicators and attitudes toward violence. Without multivariate panel models or hierarchical time-series analyses, the observed correlations cannot be interpreted as evidence supporting modernization-driven normative change.*

Response: We agree that the correlational design cannot establish causality. We have addressed this through reframing and new robustness analyses.

Reframing: The manuscript now frames the analysis as documenting associations rather than testing causal theories. The Background states:

"While correlational evidence cannot establish causality, correlations between trends provide a stronger test of hypothesized relationships than correlations in cross-sectional data. Cross-sectional correlations confound the hypothesized effect with all stable between-country differences (e.g., geography, colonial history, cultural heritage). Correlations between within-country changes over time control for these time-invariant factors, isolating the association between temporal co-movements [21]."

Several of the factors the reviewer lists as potential confounders — legal reforms, public awareness campaigns, expansion of women's rights movements — feature in parallel theories of normative change and are plausibly shaped by human development through education, media access, and institutional capacity. In cross-country work, however, the distinction between a confounder and a mechanism is rarely empirically clean: the same process may be partly enabled by development and partly driven by independent transnational forces. We therefore do not attempt to statistically partition these pathways. What we do show is that the HDI-acceptability association survives the most demanding specification we can run.

To address the request for multivariate panel modelling, the Results now add:

"To further examine the robustness of the HDI-acceptability association, we conducted two supporting analyses. First, a falsification test confirmed temporal ordering: lag correlations (HDI changes preceding acceptability changes) were negative, whereas lead correlations (HDI changes following acceptability changes) were near zero (S4 Fig). Second, we fit a multilevel longitudinal model to the wave-level data with a series of control variables (S4 Table)."

The same subsection then reports the model results:

"With HDI alone, the within-country standardised β is −0.49 (95% CI [−0.88, −0.10], p = .014) for women and −1.04 [−1.51, −0.58], p < .001 for men. Adding plausibly-exogenous lagged controls (armed-conflict intensity; ODA as % of GNI) moves the coefficients to −0.56 (W2; p = .006) and −0.91 (M2; p < .001); further adding partly-endogenous lagged controls (trade openness; women's parliamentary representation) yields −0.67 (W3; p = .007) and −1.10 (M3; p < .001). The within-country HDI effect is thus consistent across specifications and, if anything, strengthens under fuller control; the between-country HDI effect is also robustly negative for women (β = −0.41 to −0.56, all p ≤ .011) but near zero and non-significant for men."

The Limitations section acknowledges: "Unmeasured confounders such as conflict dynamics and shifts in reporting behavior could drive both development and normative change, though the Results robustness analyses constrain the most obvious alternatives."

## R1.6: HDI versus GDI interpretation may be measurement artifact

*Regarding the interpretation of the HDI versus GDI findings, I would add that warrants caution. The authors conclude that overall development appears to be more strongly associated with declining acceptance of IPVAW than improvements in gender equality. However, this conclusion may be partly an artifact of measurement. HDI and GDI are strongly correlated, and the GDI's construction as a ratio between male and female outcomes compresses variation across countries. In my view, the weaker statistical relationship observed for GDI therefore does not necessarily imply that gender equality plays a smaller role in shaping norms around violence. I therefore believe that a more nuanced approach would incorporate direct measures of gender institutions, legal protections, or female political representation rather than relying solely on development indices.*

Response: We agree and now state this caution directly in the Discussion:

"HDI tracked attitudinal change more closely than GDI across all lags and for both genders. The GDI is constructed as a ratio of female-to-male achievement, which compresses cross-country variation and likely attenuates correlations on measurement grounds. HDI and GDI are also highly correlated in our sample (r = 0.72), so their independent contributions are difficult to disentangle. Direct measures of gender-specific institutions (legal protections, female political representation) would provide a more rigorous test."

We also partly address the reviewer’s suggestion empirically by including women's parliamentary representation in the fully controlled multilevel model reported in S4 Table.

## R1.7: Attitudes-prevalence causal direction unclear

*As for the analysis linking attitudinal change to changes in the prevalence of violence, I believe it also requires more careful interpretation. The authors report a moderate correlation between trends in acceptance and trends in prevalence (approximately r = 0.35, rising to about 0.51 when an outlier country is excluded). However, the causal direction of this relationship remains unclear. Normative change may indeed influence behavior, but it is equally plausible that declining violence alters attitudes, that both are shaped by institutional reforms, or that measurement artifacts affect reported prevalence. In addition, changes in stigma surrounding domestic violence could simultaneously reduce expressed acceptance while increasing or decreasing reporting rates, thereby complicating interpretation of trend correlations.*

Response: We revised the interpretation throughout to avoid implying a causal direction. The Abstract now states: "Finally, countries with faster declines in acceptability tended to show faster reductions in physical IPVAW prevalence (r = 0.35)."

The Discussion now states: "The moderate correlation between acceptability and prevalence trends is consistent with a social-ecological understanding in which normative change is one component of a multi-level system and causal arrows may run in both directions." It also discusses the reporting issue explicitly: "As violence becomes more stigmatized, survivors in some settings may become more willing to disclose it, inflating measured prevalence even as actual violence declines, while in other settings disclosure may fall."

The Conclusion now uses the more cautious phrasing: "This study provides the first cross-national evidence that improvements in human development are associated with a global decline in the acceptability of IPVAW, and that this normative shift corresponds with reductions in experienced violence."

Table 2 likewise now uses the guarded formulation "Norm change might translate into reduced violence."

## R1.8: Social desirability bias assumption untested

*Another issue concerns potential social desirability bias. As public discourse increasingly condemns domestic violence worldwide, respondents may feel greater pressure to provide socially acceptable answers in surveys. This possibility is acknowledged but not sufficiently examined. The colleagues assume that systematic increases in social desirability bias are unlikely, yet this assumption is not empirically tested and may be unrealistic given the global expansion of gender equality campaigns during the study period.*

Response: We now address this concern directly in the Limitations subsection:

"Social desirability is a first-order concern. DHS-type direct-questioning items understate both acceptability and experience of IPVAW [35, 36]; cognitive interviews confirm frequent socially-desirable responding on such items [37], and psychometric evidence suggests this bias is driven by impression management rather than self-deception [38]. Because impression management tracks prevailing norms, this bias is likely to grow as anti-violence norms spread. Three features of our data nonetheless constrain the explanation: the acceptability–prevalence correlation uses a separate DHS module with distinct privacy protocols; the HDI association draws on an independent data source (UNDP); and HDI changes precede acceptability changes by several years, a pattern difficult to attribute to concurrent shifts in reporting norms. Convergence across measurements and sources points to genuine normative change alongside any shift in reporting."

## Response to Reviewer 2 (Roxanne Keynejad)

## R2.1: Title — consider amending to include study methods

*TITLE: It isn't immediately clear why "human development" is in the title without explictly mentioning the human development and gender development indices.; I wonder if the authors would consider amending the title to more clearly indicate the study methods (e.g. "global Demographic and Health Survey trends from 1999-2023")*

Response: We revised the title to: "Trends in the Acceptability and Prevalence of Intimate Partner Violence: Evidence from Demographic and Health Surveys, 1999–2024"

## R2.2: Abstract Line 23 — "Four years earlier" ambiguous

*ABSTRACT*

*Line 23 "Four years earlier" - than which time point? 2023?*

Response: The Abstract now uses "at a 4-year lag" instead: "We tested the association between these country-level trends and longitudinal changes in the Human Development Index (HDI) and Gender Development Index (GDI) at a 4-year lag." The Results section of the Abstract states: "Countries with faster increases in HDI tended to show faster declines in acceptability at a 4-year lag (women: r = -0.33; men: r = -0.54)."

## R2.3: Abstract Line 31 — Outlier detail too specific for abstract

*Line 31 While interesting, I suggest that the point about greater reductions when excluding a post-conflict outlier is more detail than is needed in the abstract.*

Response: We removed the outlier detail from the Abstract. The Abstract Results now state only: "countries with faster declines in acceptability tended to show faster reductions in physical IPVAW prevalence (r = 0.35)." The outlier discussion (Sierra Leone) remains in the Results section where it is appropriate.

## R2.4: Abstract Line 33 — "cross-country" terminology

*Line 33 "cross-country" - perhaps, global? Or international?*

Response: The Conclusion of the Abstract now uses "cross-national": "This study provides the first cross-national evidence that improvements in human development are associated with a global decline in the acceptability of IPVAW."

## R2.5: Abstract Line 38 — "safer social norms"

*Line 38 "safer social norms" - perhaps "gender-equitable" social norms?*

Response: The Abstract Conclusion now reads: "broad-based development strategies benefiting entire populations may be particularly effective for fostering gender-equitable social norms."

## R2.6: Background Line 60 — Summarize what DHS is before citing

*BACKGROUND*

*- Line 60 I suggest briefly summarising what the DHS is before reporting a previous finding from it.*

Response: The DHS is introduced at first mention in the Background, paragraph 2: "Throughout this paper, 'acceptability of IPVAW' refers to endorsement of the view that a husband is justified in hitting or beating his wife under at least one specified circumstance, as measured by items from the Demographic and Health Surveys (DHS), a long-running program of nationally representative household surveys conducted in low- and middle-income countries." The full description appears in the study summary paragraph: "analyzing data from the DHS, a series of nationally representative household surveys conducted across low- and middle-income countries, from 1999–2024." The Methods section provides the formal description: "This study is a secondary analysis of data from the DHS Program [22], publicly available at https://dhsprogram.com/. [...] The DHS provides comprehensive and comparable population-level data across many countries and time points."

## R2.7: Background — Define "acceptance" of IPVAW

*- It would help to briefly define "acceptance" of IPVAW. Does it refer to "acceptability" (how acceptable people consider IPVAW to be) or people's personal acceptance/tolerance/lack of opposition to IPVAW. This is explained in the methods but authors would benefit of knowing this at the Background stage.*

Response: The Background now includes an operational definition at first extended use (Background, paragraph 2): "Throughout this paper, 'acceptability of IPVAW' refers to endorsement of the view that a husband is justified in hitting or beating his wife under at least one specified circumstance, as measured by items from the Demographic and Health Surveys (DHS)." This clarifies for readers that "acceptability" refers to survey-measured endorsement of specific justifications, not personal tolerance in a broader sense. The full operationalization details (five specific contexts, binary coding) follow in the Methods.

## R2.8: Background Lines 63-65 — Current developments in US refuting development-tolerance association

*- Line 63-65 it is hard when reading this sentence not to wonder about current developments in the United States directly refuting the association between economic development and tolerance of (gender) equality. The authors might want to acknowledge this point or mention it in the Discussion.*

Response: The revised Discussion addresses this with a Limitations subsection grounded in scholarship on anti-gender movements [39–42]. It acknowledges that organized resistance to gender equality now operates at national scale and notes that our study period (1999–2024) largely predates the most intensive phase of this mobilization. The manuscript states:

"Organized backlash is a second concern. Anti-gender campaigns unite religious conservatives, right-wing populists, and opponents of “gender ideology” through legislation restricting reproductive rights, withdrawal from agreements such as the Istanbul Convention [39], and attacks on feminist organizations [40-42]. Our data (1999–2024) largely predate the most intensive phase of this mobilization, so the observed trends may not extrapolate to the current political landscape."

## R2.9: Background — Move Figure 1 to Methods

*- I suggest moving Figure 1 to the Methods section.*

Response: We have moved Figure 1 from the Background to the Methods section, placing it in the Methods section before the Covariates for Robustness Analyses subsection. We reframed the caption to emphasize the figure’s analytic function: ‘Analytic framework. Arrows indicate the hypothesized relationships tested in this study: human development (HDI, GDI) is associated with trends in the acceptability of IPVAW, and trends in acceptability are associated with trends in prevalence.’ A forward reference in the Background now directs readers to the Methods section for the full conceptual model.

## R2.10: Background — Can the "correlations between trends" claim be referenced?

*- Can the statement, "correlations between trends provide a stronger test of hypothesized relationships than correlations in cross-sectional data" be referenced and/or explained, briefly?*

Response: The original manuscript stated this claim without elaboration. We expanded it with a brief explanation of why trend correlations provide a stronger test, and added a supporting reference [21]:

"While correlational evidence cannot establish causality, correlations between trends provide a stronger test of hypothesized relationships than correlations in cross-sectional data. Cross-sectional correlations confound the hypothesized effect with all stable between-country differences (e.g., geography, colonial history, cultural heritage). Correlations between within-country changes over time control for these time-invariant factors, isolating the association between temporal co-movements [21]."

## R2.11: Methods — Define "trend" statistically

*METHODS*

*Could the term "trend" be briefly statistically defined, to help less familiar readers understand its statistical meaning over and above the lay understanding of the word?*

Response: The Methods section now includes an explicit definition: "Throughout this paper, a 'trend' refers to the estimated annual rate of change in a variable over the study period for a given country, measured in percentage points per year."

## R2.12: Methods Line 240 — Move AI disclosure to end of manuscript

*Line 240 I believe that the statement about use of AI (Claude) should be at the end of the manuscript rather than in the methods section.*

Response: We moved the AI disclosure to a section at the end of the manuscript, after Acknowledgements:

"Claude (Anthropic) was used to assist with text editing, literature searches, and drafting supplementary statistical analyses during manuscript preparation and revision. All AI-generated content was reviewed, verified, and approved by the authors, who take full responsibility for the final manuscript."

## R2.13: Results Lines 249-251 — "Pierotti period" opaque

*RESULTS*

*Line 249-251 Since many readers will not be deeply familiar with the Pierotti study, the phrasing of "Pierotti period" and "post-Pierotti period" would be better stated simply using the years being referred to.*

Response: The Results now use specific year ranges instead of "Pierotti period": "for the 21 countries included in Pierotti's original study [11], the decrease in the acceptability of IPVAW among women continued robustly (the mean decrease was 1.82 percentage points per year in the earlier period (1999–2010) and 0.91 percentage points per year in the later period (2011–2024))."

## R2.14: Results — Use "acceptability of IPV among women/men"

*Throught the manuscript, it would be clearer to say "the acceptability of IPV among women/men" rather than "women/men's acceptance of..." because "acceptance of" sounds like their readiness to personally accept IPV (for readers who may not have read the Methods section).*

Response: We adopted this suggestion for clarity. As Reviewer 2 noted, "acceptability of IPVAW among women/men" avoids the implication that respondents personally accept violence, making the phrasing clearer for readers unfamiliar with the DHS methodology. We have adopted "acceptability" consistently throughout the manuscript — in the title, Abstract, Background, Methods, Results, and Discussion — whenever referring to this construct.

## R2.15: Results Line 289 — "Consistent with modernization theory" is interpretation

*Line 289 "Consistent with modernization theory" - this is an interpretation that would be more helpful in the Discussion.*

Response: We removed interpretive statements from the Results section. The Results section "Association with Human Development and Gender Equity" now reports findings without theoretical interpretation. The modernization theory interpretation appears only in the Discussion subsection "Development and normative change."

## R2.16: Results Lines 299-302 — Repeats Methods content

*Line 299-302 repeats content in the Methods.*

Response: We removed methodological details from Results. The Results section reports findings directly, with the full methodological description contained in the Methods section.

## R2.17: Results Lines 305-308 — Interpretations belong in Discussion

*Line 305-308 "suggests that..." these interpretations would fit better in the Discussion.*

Response: We moved interpretive statements from Results to Discussion. The Results section now presents findings without editorial commentary about their implications.

## R2.18: Results Lines 318-319 — "Is consistent with..." belongs in Discussion

*Line 318-319 "Is consistent with..." would be better in the Discussion.*

Response: The Results section no longer contains "consistent with" interpretive framing. Results are reported as observed patterns; their theoretical consistency is discussed in the Discussion section.

## R2.19: Results Lines 321-324 — Sierra Leone detail belongs in Discussion

*Line 321-324 the additional detail about Sierra Leone would be better in the Discussion.*

Response: The Results section retains a brief factual mention of Sierra Leone as a statistical outlier: "Visual inspection of Figure 4 reveals Sierra Leone (SLE) as an outlier, the only country with a strong increase in the prevalence of IPVAW." The contextual explanation (post-conflict disruption) appears in the Discussion section "From norms to behavior": "Post-conflict contexts (such as Sierra Leone in our sample) can show rising violence linked to social disruption despite declining acceptability."

## R2.20: Table 2 — Non-data column could fit in Discussion

*Table 1 does not have a top line with labels for each column. The third column is also not information deduced from the data so this content could fit better in the Discussion.*

Response: We agree that this concern applies to Table 2 (Summary of Key Findings and Policy Implications), whose "Policy implication" column contains interpretive content rather than empirical results. We have moved Table 2, together with the Discussion "Policy implications" subsection, from Results to Discussion, where the policy framing is more appropriate. Table 1 (correlation matrix) now includes column headers as well.

## R2.21: Discussion — Too long, repetition of results

*DISCUSSION*

*This section is very interesting to read but rather long in relation to the rest of the manuscript. Some of the repetition of the results could be reduced.*

Response: We tightened the Discussion by reducing restatement of results. Results are referenced concisely (e.g., citing specific correlation values) rather than re-described at length. The Discussion is organized around four labeled subsections, each building on the reported findings without restating them in full.

## R2.22: Discussion Lines 355-357 — Public health campaigns claim premature

*Line 355-357 It does not necessarily follow that public health campagins will influence acceptability. This point is elaborated later so can be removed here.*

Response: We removed anticipatory claims about public health campaigns. The Policy Implications subsection frames findings in terms of evidence-based recommendations rather than predictions, using language such as "suggest that" and "may."

## R2.23: Discussion Line 390 — Explain "exogenous global cultural script diffusion model"

*Line 390 Please explain what the "exogenous \"global cultural script\" diffusion model" comprises.*

Response: The concept is now explained in both the Background and Discussion. The Background paragraph states: "Pierotti, analyzing DHS data from 26 countries, found that structural changes within countries—rising urbanization, education, and media access—could not account for the decline in IPVAW acceptability; she attributed the trend to the diffusion of a global cultural script condemning domestic violence, transmitted through international organizations, media, and education systems [11]. Swindle extended this by showing that mass media exposure is a primary conduit for normative diffusion, with media access predicting individual-level attitude change [15]." The Discussion refers back to this as the "“global cultural script” diffusion model advanced by Pierotti [11] and Swindle [15]."

## R2.24: Discussion Line 564 — Explain what RESPECT is

*Line 564 it would help to say something about what RESPECT is.*

Response: The revised manuscript provides a full description: "WHO's RESPECT [34], an evidence-based guide for preventing violence against women organized around seven strategies: Relationship skills strengthening, Empowerment of women, Services ensured, Poverty reduced, Enabling environments, Child and adolescent abuse addressed, and Transformed attitudes, beliefs, and norms (WHO 2019, updated 2025)."

## R2.25: Discussion — Patriarchal backlash, HDI and populist misinformation

*The authors only fleetingly acknowledge patriarchal backlashes, which by now are no longer localised matters but being implemented on a national scale in many countries. Can the authors acknowledge this and comment on how their proposal that investment in HDI components might serve to diminish support for populist misinformation as living conditions improve?*

Response: The revised Discussion includes a Limitations subsection addressing this concern, citing recent scholarship on anti-gender mobilization [39–42]. It acknowledges that organized resistance to gender equality now operates at national scale, while noting that our data (1999–2024) largely predate the most intensive phase. The manuscript states:

"Organized backlash is a second concern. Anti-gender campaigns unite religious conservatives, right-wing populists, and opponents of “gender ideology” through legislation restricting reproductive rights, withdrawal from agreements such as the Istanbul Convention [39], and attacks on feminist organizations [40-42]. Our data (1999–2024) largely predate the most intensive phase of this mobilization, so the observed trends may not extrapolate to the current political landscape."

## R2.26: Minor — Consistent acronym usage

*MINOR POINTS*

*- It would help to be consistent in the use of acronyms. IPVAW is mostly used but IPV appears on lines 55, 86, 94, 114 and then "intimate partner violence" is said in words on lines 129, 147, 246, 356, 372, 487, 534.*

Response: We revised the manuscript to use "IPVAW" consistently throughout.

## R2.27: Minor — Define all acronyms at first use, don't redefine

*- Please define all acronyms at first use e.g. Line 60 MICS, Line 69 GDP, Line 74 HDI, line 200 OLS, line 237 ICF, IRB. It would also help not to redefine acronyms later, e.g. DHS on line 108, HDI on line 288, IPVAW on line 343-4, SDG on line 537.*

Response: Acronyms have been reviewed and expanded where needed. IPVAW is defined in the first sentence of the Background; DHS is defined at first mention; HDI, GDI, SDG, WHO, and OLS are each expanded at first use. We removed redundant redefinitions.

## R2.28: Minor — "through 2022" unclear

*- Line 60 "through 2022" is not a phrase all English speakers would understand. I suggest, "up to" or "until" 2022.*

Response: The phrase "through 2022" no longer appears in the manuscript. The data coverage is described as "from 1999–2024" or with specific date ranges where needed.

## R2.29: Minor — State "years" after ages

*- Line 119 I suggest stating "years" after all ages.*

Response: Done. The manuscript now reads "aged 15–49 years" throughout.

## R2.30: Minor — "we don't know which lag to expect" too casual

*- Line 214 "we don't know which lag to expect" is a little casual.*

Response: We replaced the informal phrasing. The Methods section now reads: "a priori, the lag between development improvements and normative change is theoretically uncertain, and no prior empirical work specifies an expected duration."

## R2.31: Minor — Voice inconsistency (active vs passive)

*LInes 211-233 there is switching from active voice ("we correlated...") to passive voice ("analysis was repeated.." Consistency would help.*

Response: The Methods section uses consistent active voice: "We compiled all available DHS datasets," "We calculated the weighted percentage," "We measured women's experiences," "We obtained Human Development Index (HDI) data," "We used Pearson correlation coefficients."

## R2.32: Minor — "not perfect synchrony" informal

*Line 280 and line 360 "not perfect synchrony" could be phrased more formally.*

Response: The revised text uses "not complete synchrony": "This moderate-to-strong correlation indicates substantial consistency in how acceptability is changing across genders within countries, though not complete synchrony."

## R2.33: Minor — "wives" should be "women"

*Line 354 I suggest "women" rather than "wives".*

Response: We replaced "wives" with "women" except where it appears in direct quotations of historical legal language or DHS survey item wording. The one remaining instance in the main text refers to 1870s legal history: "most US states had renounced men's right to physically discipline their wives by the late 1870s." The DHS questions themselves use "wife" in their standard wording, and this is preserved when describing the survey items.

## R2.34: Data Availability Statement

*Have the authors made all data underlying the findings in their manuscript fully available (please refer to the Data Availability Statement at the start of the manuscript PDF file)?*

*Reviewer #2: Yes [but see Data Availability Statement comment]*

*Note: Reviewer 2 flagged that the Data Availability Statement was not PLOS-compliant.*

Response: The revised Data Availability statement now specifies all data sources and access procedures:

*“*The aggregated country-year dataset and all analysis code needed to reproduce the reported findings are archived at <https://doi.org/10.17605/OSF.IO/YU3F7> (OSF). The underlying DHS individual-level datasets are third-party and available from the DHS Program (<https://dhsprogram.com>) upon registration. HDI and GDI data are public from UNDP (<https://hdr.undp.org>). Covariates used in the robustness analyses were obtained from the World Bank World Development Indicators, <https://data.worldbank.org>.”
