# Supplementary material for: Trends in the acceptability and prevalence of intimate partner violence: Evidence from Demographic and Health Surveys, 1999–2024
Source: PLOS Glob Public Health. 2026 Jun 24;6(6):e0006628. doi: 10.1371/journal.pgph.0006628 (PMC13293389; doi:10.1371/journal.pgph.0006628)
Supplement: Attachment — Submitted filename: Response_to_Editor_R2_2026-05-17.docx [file pgph.0006628.s004.docx]

# Response to Editor — PGPH-D-26-00404 (2^nd^ revision)

Dear Dr. Zeinali,

Thank you for the encouraging assessment of our revision and for the five concrete points raised in the minor-revision decision. Below we describe how each has been addressed. Quoted passages are taken verbatim from the revised manuscript.

## 1. Causal and policy language across the manuscript

We revised the Abstract, Discussion, Policy Implications, and Conclusion to ensure that the wording remains consistently associational and does not imply causal or policy-relevant inferences beyond what a country-level correlational design can support.

The Abstract now closes:

Although the association with overall development was somewhat stronger than with gender-specific equity, this difference is also consistent with measurement properties of the GDI and the high HDI–GDI correlation in our sample, and should be interpreted cautiously rather than as evidence that broad-based strategies are more effective. These cross-national associations are consistent with a role for comprehensive development investments in education, health, and economic prosperity, though the design cannot establish that such investments cause reductions in violence against women.

The Conclusion now reads:

Broad-based initiatives raising living standards, health, and education are associated with normative change toward rejection of domestic violence; whether such initiatives causally contribute to reductions in violence prevalence remains to be established by designs that can support causal inference.

Parallel softenings have been applied to the Discussion (“From norms to behavior” and the four-findings summary) and to Table 2’s policy-implication column, which now frames implications as consistent with rather than demonstrated by the evidence.

## 2. HDI versus GDI comparison

We further tempered the HDI–GDI contrast in both the Abstract (quoted above) and the Discussion. The Discussion paragraph on this comparison now closes:

Given these measurement caveats, our results should not be read as evidence that broad development matters more than gender-equitable development for normative change. The pattern is consistent with both processes operating jointly.

The earlier wording in the same paragraph also flags the GDI’s compressed cross-country variation and the high HDI–GDI correlation (r = 0.72) as measurement-side explanations for the gap, before any substantive reading is offered.

## 3. Assertive Discussion passages and the historical argument

The historical material contrasting endogenous and exogenous explanations is now presented as illustrative rather than dispositive:

Historical examples are illustrative rather than dispositive. Most US states renounced men’s right to physically discipline their wives by the late 1870s, and the UK followed in 1878. These cases suggest that internal development processes can contribute to normative change alongside international diffusion, but observational data of this kind cannot adjudicate between the two pathways.

The “Four findings” paragraph at the top of the Discussion was tightened: re-quoted Results numbers were removed, and the HDI–GDI gap is now flagged inline with a pointer to the measurement caveats that follow. The “From norms to behavior” subsection lost its trailing policy clause so that it reads as a description of the correlation rather than a recommendation.

## 4. Limitations: neutral on mechanisms versus confounders

The Limitations opener was rewritten to treat mediation and confounding as parallel rather than ranking them:

Our design is correlational. Several factors connected to the HDI–acceptability association, including legal reforms, awareness campaigns, and women’s rights movements, may operate as mechanisms that transmit development effects, as confounders independent of development, or as both. Because we cannot empirically distinguish these roles, we do not control for these factors in the main analysis, and readers should weigh the evidence accordingly.

We also rewrote the social-desirability paragraph and the standard-limitations paragraph in this section. The social-desirability text now reframes rather than rebuts the bias concern: we acknowledge that part of the measured decline may reflect a shift in public rather than private acceptance, and we argue, drawing on the social-norms literature, that public norms are themselves behaviorally consequential through community sanctions. The standard-limitations paragraph now includes an explicit acknowledgement of residual country-level confounders alongside the mechanism caveat:

As a country-level analysis, we cannot identify the specific mechanisms (individual education, community norms, legal reforms) through which development affects attitudes, nor fully rule out unmeasured country-level confounders beyond those addressed in the robustness analyses.

## 5. Tightening of Discussion and Policy Implications

The Discussion was tightened by removing re-quotation of Results numbers and by trimming the closing clauses of two subsections that had drifted into policy advocacy.

The Policy Implications section was rewritten in proportion to the evidence:

Two readings follow, both proportionate to the correlational design. First, the cross-national pattern is consistent with HDI-aligned investments in education (SDG 4), health (SDG 3), and economic opportunity having relevance for both human welfare and norms about violence (SDG 5), with any normative correlate of development investments emerging over a medium-term horizon rather than immediately.

Table 2’s implication column was reworded in parallel; the previous “may yield dual benefits for human welfare and violence prevention” row 3 now reads “Pattern is consistent with development investments being relevant to both human welfare and normative change; causal inference about violence prevention requires interventional evidence.”

We hope these revisions address each of the five points to the editor’s satisfaction.

Sincerely,

Kimmo Eriksson, corresponding author
