# Supplementary material for: Trends in the acceptability and prevalence of intimate partner violence: Evidence from Demographic and Health Surveys, 1999–2024
Source: PLOS Glob Public Health. 2026 Jun 24;6(6):e0006628. doi: 10.1371/journal.pgph.0006628 (PMC13293389; doi:10.1371/journal.pgph.0006628)
Supplement: Peer review history

# Peer review: Decision Letter 0

**DOI:** 10.1371/journal.pgph.0006628.r001

**Authors:** Zahra Zeinali

26 Mar 2026

PGPH-D-26-00404

Human Development and the Acceptance and Prevalence of Intimate Partner Violence: A Global Analysis of Temporal Trends

PLOS Global Public Health

Dear Dr. Eriksson,

Thank you for submitting your manuscript to PLOS Global Public Health. After careful consideration, we feel that it has merit but does not fully meet PLOS Global Public Health’s publication criteria as it currently stands. Therefore, we invite you to submit a revised version of the manuscript that addresses the points raised during the review process.

Please submit your revised manuscript by 30 April 2026. If you will need more time than this to complete your revisions, please reply to this message or contact the journal office at globalpubhealth@plos.org. When you're ready to submit your revision, log on to https://www.editorialmanager.com/pgph/ and select the 'Submissions Needing Revision' folder to locate your manuscript file.

Please include the following items when submitting your revised manuscript:

- A letter that responds to each point raised by the editor and reviewer(s). You should upload this letter as a separate file labeled 'Response to Reviewers'.
- A marked-up copy of your manuscript that highlights changes made to the original version. You should upload this as a separate file labeled 'Revised Manuscript with Track Changes'.
- An unmarked version of your revised paper without tracked changes. You should upload this as a separate file labeled 'Manuscript'.

Guidelines for resubmitting your figure files are available below the reviewer comments at the end of this letter.

We look forward to receiving your revised manuscript.

Kind regards,

Zahra Zeinali, MD MPH DrPH

Academic Editor

PLOS Global Public Health

Journal Requirements:

If the reviewer comments include a recommendation to cite specific previously published works, please review and evaluate these publications to determine whether they are relevant and should be cited. There is no requirement to cite these works unless the editor has indicated otherwise.

Additional Editor Comments (if provided):

Thank you for submitting your manuscript, “Human Development and the Acceptance and Prevalence of Intimate Partner Violence: A Global Analysis of Temporal Trends,” to PLOS Global Public Health.

Your manuscript addresses an important and policy-relevant question and draws on an extensive cross-national dataset. The reviewers and I agree that the paper has potential. In particular, the descriptive evidence suggesting declining acceptability of intimate partner violence against women across many settings is of interest. However, substantial revision is required before the manuscript can be reconsidered for publication.

After considering the reviewer reports and my own assessment, I am inviting you to submit a major revision.

The principal concerns relate to the alignment between the manuscript’s theoretical framing, analytic approach, and conclusions. At present, the paper relies heavily on modernization theory as the main explanatory framework, but the analysis does not adequately engage with alternative explanations, including global norm diffusion, institutional diffusion, or transnational advocacy processes. More importantly, the empirical strategy does not permit clear adjudication between these mechanisms. The revised manuscript should therefore either broaden and rebalance the theoretical framing or more clearly position the analysis as descriptive and correlational rather than explanatory.

A second major issue concerns the statistical and methodological approach. The current strategy of estimating country trends using first and last survey observations is simple and intuitive, but it discards intermediate observations and may obscure non-linear trends. In addition, the core analyses rely primarily on bivariate correlations of country-level trend estimates, which limits the strength of the inferences that can be drawn. The revised manuscript should strengthen the analytic approach where possible, provide clearer robustness analyses using all available time points, and temper the interpretation of the findings accordingly.

The manuscript also requires a more critical treatment of measurement. The operationalization of “acceptance” of IPVAW using the DHS item set should be discussed more carefully, including its conceptual limits, its binary construction, and the omission of certain commonly cited justifications such as infidelity. Relatedly, the discussion of social desirability bias and reporting bias should be expanded, particularly given the manuscript’s interpretation of temporal change in both attitudes and prevalence.

The interpretation of the comparative HDI and GDI findings also needs greater caution. As currently written, the manuscript risks overstating the conclusion that overall human development matters more than gender equality. Differences in observed associations may reflect measurement properties of these indices rather than substantive differences in explanatory importance.

Finally, the section linking change in attitudinal acceptance to change in violence prevalence should be interpreted more cautiously. The association is potentially important, but the direction of causality remains unclear and alternative explanations remain plausible.

In preparing your revision, please address the following:

strengthen and rebalance the theoretical framing, including engagement with alternative explanatory perspectives;

revise the manuscript to ensure that causal language does not exceed what can be supported by the design;

strengthen or more transparently justify the analytic strategy, including robustness analyses using all available survey waves;

expand the discussion of measurement limitations, including the construction and cross-context validity of the attitudinal measure;

address social desirability and reporting bias more fully;

interpret the HDI versus GDI findings more cautiously;

moderate the interpretation of the association between attitudes and prevalence; and

revise the manuscript for clarity, concision, consistency of terminology, table presentation, and standard English throughout.

Please also address the reviewers’ specific line-by-line and presentation-related comments in full, including comments on the title, abstract, acronyms, structure of the Results and Discussion, and table formatting. In addition, please ensure that the Data Availability Statement and underlying materials fully comply with journal policy.

When submitting your revision, please include a detailed response to the reviewers and a marked-up version of the manuscript showing all changes.

A decision of major revision does not guarantee acceptance, and the revised manuscript may be returned for further review. However, I would be willing to reconsider a substantially revised version that addresses the concerns above.

[Note: HTML markup is below. Please do not edit.]

Reviewers' comments:

Reviewer's Responses to Questions

**Comments to the Author**

1. Does this manuscript meet PLOS Global Public Health’s publication criteria? Is the manuscript technically sound, and do the data support the conclusions? The manuscript must describe methodologically and ethically rigorous research with conclusions that are appropriately drawn based on the data presented.

Reviewer #1: Partly

Reviewer #2: Yes

**********

2. Has the statistical analysis been performed appropriately and rigorously?

Reviewer #1: No

Reviewer #2: Yes

**********

3. Have the authors made all data underlying the findings in their manuscript fully available (please refer to the Data Availability Statement at the start of the manuscript PDF file)?

The PLOS Data policy requires authors to make all data underlying the findings described in their manuscript fully available without restriction, with rare exception. The data should be provided as part of the manuscript or its supporting information, or deposited to a public repository. For example, in addition to summary statistics, the data points behind means, medians and variance measures should be available. If there are restrictions on publicly sharing data—e.g. participant privacy or use of data from a third party—those must be specified.

Reviewer #1: No

Reviewer #2: Yes

**********

4. Is the manuscript presented in an intelligible fashion and written in standard English?

PLOS Global Public Health does not copyedit accepted manuscripts, so the language in submitted articles must be clear, correct, and unambiguous. Any typographical or grammatical errors should be corrected at revision, so please note any specific errors here.

Reviewer #1: No

Reviewer #2: Yes

**********

5. Review Comments to the Author

Please use the space provided to explain your answers to the questions above. You may also include additional comments for the author, including concerns about dual publication, research ethics, or publication ethics. (Please upload your review as an attachment if it exceeds 20,000 characters)

Reviewer #1: The manuscript entitled “Human Development and the Acceptance and Prevalence of Intimate Partner Violence: A Global Analysis of Temporal Trends” addresses an important and timely question: whether improvements in human development are associated with declining social acceptance of intimate partner violence against women (IPVAW) and whether changes in norms correspond with changes in the prevalence of violence. My colleagues use Demographic and Health Survey data covering nearly 1.9 million women and more than half a million men across 69 countries between 1999 and 2023, which provides a remarkably large and geographically diverse empirical basis for analysis! Their manuscript therefore engages with a central issue in the global gender equality literature and has the potential to contribute to ongoing debates on the macro-level drivers of normative change regarding violence against women.

The authors’ central empirical claim is that acceptance of IPVAW has declined in most countries examined and that this decline is associated with improvements in the Human Development Index (HDI) and, to a lesser extent, the Gender Development Index (GDI). Furthermore, the authors report a moderate correlation between declining acceptance and declining prevalence of physical IPVAW. While these findings are potentially interesting, the study in its current form suffers from several theoretical, conceptual, and methodological weaknesses that significantly limit the strength of its conclusions.

In my opinion, the most fundamental limitation concerns theoretical framing. The authors rely heavily on modernization theory to interpret the observed associations between development and normative change. However, I find the engagement with the theory to be limited and insufficiently critical. The colleagues, on the one hand, present modernization as the primary explanatory framework, while, on the other hand, they only briefly mention and without substantive engagement, alternative perspectives, - such as global cultural diffusion, institutional diffusion through international organizations, transnational feminist advocacy networks, or the theory of the world polity. As a result, their theoretical discussion seems somewhat confirmatory rather than truly explanatory. Their analysis is structured in a way that implicitly assumes development-driven value change rather than rigorously testing competing explanations for the observed trends.

I wish to emphasize that this issue is compounded by the fact that the empirical strategy cannot convincingly distinguish between endogenous development processes and exogenous norm diffusion. In my view, both processes could plausibly produce the same empirical pattern: countries experiencing improvements in development indicators might simultaneously be more exposed to global human rights discourses, international development programs, and media narratives condemning domestic violence. I would like to draw the attention of colleagues to the fact that without incorporating variables capturing global institutional diffusion or policy adoption, the analysis risks attributing causal explanatory power to development indicators that may simply correlate with broader processes of globalization and norm institutionalization.

A second major concern relates to the measurement of the key attitudinal variable. If I understood correctly, the operationalization of “acceptance of intimate partner violence” relies on a composite indicator derived from five DHS questions asking whether a husband is justified in hitting or beating his wife in specific circumstances (e.g., burning food, arguing, going out without informing him). Respondents are coded as accepting violence if they endorse any of these scenarios. Here of course, although this operationalization follows established practice in DHS-based research, I find that it is conceptually blunt. It conflates very different normative positions into a single binary measure and does not capture gradations in moral justification or contextual reasoning. Furthermore, as the authors acknowledge, the DHS questions omit infidelity-related justifications, which are widely documented as among the most accepted triggers for partner violence in many societies. Consequently, do not colleagues believe that the indicator likely underestimates overall normative acceptance and may also vary in validity across cultural contexts?

The methodological strategy used to estimate temporal trends is also problematic. If again I have understood correctly, the authors calculate country-specific trends using only the first and last available survey observations, dividing the difference by the time interval between them. But, while this approach produces a simple measure of average change, it ignores all intermediate data points and assumes linear change over time. In my opinion, in countries with multiple DHS waves, this decision results in a substantial loss of information and prevents the detection of non-linear dynamics, reversals, or acceleration in normative change. The authors briefly state that results are “virtually identical” when using OLS estimates based on all available time points, but these alternative analyses are not presented in sufficient detail to allow readers to assess their robustness.

Although I am not a statistician and in general speaking, I find that the statistical design remains relatively weak for the type of causal interpretation suggested in the discussion. The core analyses rely on simple Pearson correlations between country-level trend estimates. While the authors introduce temporal lags between development indicators and attitudinal change, the analysis remains essentially correlational and does not control for potential confounders. I invite colleagues to reflect on how many factors (including legal reforms, public awareness campaigns, expansion of women’s rights movements, conflict dynamics, or shifts in reporting behavior), could simultaneously influence both development indicators and attitudes toward violence. Without multivariate panel models or hierarchical time-series analyses, the observed correlations cannot be interpreted as evidence supporting modernization-driven normative change.

Regarding the interpretation of the HDI versus GDI findings, I would add that warrants caution. The authors conclude that overall development appears to be more strongly associated with declining acceptance of IPVAW than improvements in gender equality. However, this conclusion may be partly an artifact of measurement. HDI and GDI are strongly correlated, and the GDI’s construction as a ratio between male and female outcomes compresses variation across countries. In my view, the weaker statistical relationship observed for GDI therefore does not necessarily imply that gender equality plays a smaller role in shaping norms around violence. I therefore believe that a more nuanced approach would incorporate direct measures of gender institutions, legal protections, or female political representation rather than relying solely on development indices.

As for the analysis linking attitudinal change to changes in the prevalence of violence, I believe it also requires more careful interpretation. The authors report a moderate correlation between trends in acceptance and trends in prevalence (approximately r = 0.35, rising to about 0.51 when an outlier country is excluded). However, the causal direction of this relationship remains unclear. Normative change may indeed influence behavior, but it is equally plausible that declining violence alters attitudes, that both are shaped by institutional reforms, or that measurement artifacts affect reported prevalence. In addition, changes in stigma surrounding domestic violence could simultaneously reduce expressed acceptance while increasing or decreasing reporting rates, thereby complicating interpretation of trend correlations.

Another issue concerns potential social desirability bias. As public discourse increasingly condemns domestic violence worldwide, respondents may feel greater pressure to provide socially acceptable answers in surveys. This possibility is acknowledged but not sufficiently examined. The colleagues assume that systematic increases in social desirability bias are unlikely, yet this assumption is not empirically tested and may be unrealistic given the global expansion of gender equality campaigns during the study period.

Despite these concerns of mine, I find that the manuscript has several strengths. The dataset is extensive, the research question is relevant, and the attempt to connect macro-development indicators with shifts in social norms is intellectually valuable. The descriptive evidence for a widespread decline in the acceptance of IPVAW across multiple regions is itself an important empirical contribution. However, the analytical framework and methodological design are not yet sufficiently developed to support the stronger causal claims advanced in the discussion.

In its current form, the manuscript reads more as a descriptive cross-national trend analysis than as a rigorous test of competing theories of normative change. To reach the standards expected of a serious public health journal, as it is PGPH, the paper would require substantial revision. In particular, the authors would need: a) to expand the theoretical framework, b) incorporate alternative explanatory mechanisms, c) employ more rigorous panel-based statistical models, and d) provide a deeper discussion of measurement limitations and potential reporting biases.

For these reasons, my recommendation would be major revision. The paper addresses an important topic and draws on a valuable dataset, but significant conceptual and methodological improvements are necessary before the conclusions can be considered robust.

Reviewer #2: Thank you for the opportunity to review this interesting manuscript on an important subject. I enjoyed reading this article, which is well-written and compellingly written. My primary feedback is the need to directly engage with recent patriarchal backlash and how the findings can be used to develop strategies to mitigate for anti-equity misinformation. I make some suggestions below to enhance clarity and would be interested to review a revision.

TITLE: It isn't immediately clear why "human development" is in the title without explictly mentioning the human development and gender development indices.; I wonder if the authors would consider amending the title to more clearly indicate the study methods (e.g. "global Demographic and Health Survey trends from 1999-2023")

ABSTRACT

Line 23 "Four years earlier" - than which time point? 2023?

Line 31 While interesting, I suggest that the point about greater reductions when excluding a post-conflict outlier is more detail than is needed in the abstract.

Line 33 "cross-country" - perhaps, global? Or international?

Line 38 "safer social norms" - perhaps "gender-equitable" social norms?

BACKGROUND

- Line 60 I suggest briefly summarising what the DHS is before reporting a previous finding from it.

- It would help to briefly define "acceptance" of IPVAW. Does it refer to "acceptability" (how acceptable people consider IPVAW to be) or people's personal acceptance/tolerance/lack of opposition to IPVAW. This is explained in the methods but authors would benefit of knowing this at the Background stage.

- Line 63-65 it is hard when reading this sentence not to wonder about current developments in the United States directly refuting the association between economic development and tolerance of (gender) equality. The authors might want to acknowledge this point or mention it in the Discussion.

- I suggest moving Figure 1 to the Methods section.

- Can the statement, "correlations between trends provide a stronger test of hypothesized relationships than correlations in cross-sectional data" be referenced and/or explained, briefly?

METHODS

Could the term "trend" be briefly statistically defined, to help less familiar readers understand its statistical meaning over and above the lay understanding of the word?

Line 240 I believe that the statement about use of AI (Claude) should be at the end of the manuscript rather than in the methods section.

RESULTS

Line 249-251 Since many readers will not be deeply familiar with the Pierotti study, the phrasing of "Pierotti period and "post-Pierotti period" would be better stated simply using the years being referred to.

Throught the manuscript, it would be clearer to say "the acceptability of IPV among women/men" rather than "women/men's acceptance of..." because "acceptance of" sounds like their readiness to personally accept IPV (for readers who may not have read the Methods section).

Line 289 "Consistent with modernization theory" - this is an interpretation that would be more helpful in the Discussion.

Line 299-302 repeats content in the Methods.

Line 305-308 "suggests that..." these interpretations would fit better in the Discussion.

Line 318-319 "Is consistent with..." would be better in the Discussion.

Line 321-324 the additional detail about Sierra Leone would be better in the Discussion.

Table 1 does not have a top line with labels for each column. The third column is also not information deduced from the data so this content could fit better in the Discussion.

DISCUSSION

This section is very interesting to read but rather long in relation to the rest of the manuscript. Some of the repetition of the results could be reduced.

Line 355-357 It does not necessarily follow that public health campagins will influence acceptability. This point is elaborated later so can be removed here.

Line 390 Please explain what the "exogenous "global cultural script" diffusion model" comprises.

Line 564 it would help to say something about what RESPECT is.

The authors only fleetingly acknowledge patriarchal backlashes, which by now are no longer localised matters but being implemented on a national scale in many countries. Can the authors acknowledge this and comment on how their proposal that investment in HDI components might serve to diminish support for populist misinformation as living conditions improve?

MINOR POINTS

- It would help to be consistent in the use of acronyms. IPVAW is mostly used but IPV appears on lines 55, 86, 94, 114 and then "intimate partner violence" is said in words on lines 129, 147, 246, 356, 372, 487, 534.

- Please define all acronyms at first use e.g. Line 60 MICS, Line 69 GDP, Line 74 HDI, line 200 OLS, line 237 ICF, IRB. It would also help not to redefine acronyms later, e.g. DHS on line 108, HDI on line 288, IPVAW on line 343-4, SDG on line 537.

- Line 60 "through 2022" is not a phrase all English speakers would understand. I suggest, "up to" or "until" 2022.

- Line 119 I suggest stating "years" after all ages.

- Line 214 "we don't know which lag to expect" is a little casual.

LInes 211-233 there is switching from active voice ("we correlated...") to passive voice ("analysis was repeated.." Consistency would help.

Line 280 and line 360 "not perfect synchrony" could be phrased more formally.

Line 354 I suggest "women" rather than "wives".

**********

6. PLOS authors have the option to publish the peer review history of their article (what does this mean?). If published, this will include your full peer review and any attached files.

**Do you want your identity to be public for this peer review?** If you choose “no”, your identity will remain anonymous but your review may still be made public.

For information about this choice, including consent withdrawal, please see our Privacy Policy.

Reviewer #1: No

Reviewer #2: **Yes:** Roxanne Keynejad

**********

[NOTE: If reviewer comments were submitted as an attachment file, they will be attached to this email and accessible via the submission site. Please log into your account, locate the manuscript record, and check for the action link "View Attachments". If this link does not appear, there are no attachment files.]

-->Figure Resubmissions:

While revising your submission, we strongly recommend that you use PLOS’s NAAS tool (https://ngplosjournals.pagemajik.ai/artanalysis) to test your figure files. NAAS can convert your figure files to the TIFF file type and meet basic requirements (such as print size, resolution), or provide you with a report on issues that do not meet our requirements and that NAAS cannot fix.

After uploading your figures to PLOS’s NAAS tool - https://ngplosjournals.pagemajik.ai/artanalysis, NAAS will process the files provided and display the results in the "Uploaded Files" section of the page as the processing is complete. If the uploaded figures meet our requirements (or NAAS is able to fix the files to meet our requirements), the figure will be marked as "fixed" above. If NAAS is unable to fix the files, a red "failed" label will appear above. When NAAS has confirmed that the figure files meet our requirements, please download the file via the download option, and include these NAAS processed figure files when submitting your revised manuscript.

# Author comment: Author response to Decision Letter 1

**DOI:** 10.1371/journal.pgph.0006628.r002

23 Apr 2026

Attachment Submitted filename: renamed_2a1d4.docx

# Editor report: Decision Letter 1

**DOI:** 10.1371/journal.pgph.0006628.r003

**Authors:** Zahra Zeinali

1 May 2026

PGPH-D-26-00404R1

Trends in the Acceptability and Prevalence of Intimate Partner Violence: Evidence from Demographic and Health Surveys, 1999-2024

PLOS Global Public Health

Dear Dr. Eriksson,

Thank you for submitting your manuscript to PLOS Global Public Health. After careful consideration, we feel that it has merit but does not fully meet PLOS Global Public Health’s publication criteria as it currently stands. Therefore, we invite you to submit a revised version of the manuscript that addresses the points raised during the review process.

Thank you for the substantial revisions made in response to the previous decision. The manuscript is notably improved. In particular, I appreciate the clearer framing of the attitudinal construct as acceptability, the strengthened engagement with alternative theoretical perspectives beyond modernization theory, the expanded discussion of measurement limitations and social desirability bias, the improved data availability and reproducibility statement, and the addition of robustness analyses addressing the earlier methodological concerns.

The paper is now much stronger and closer to being suitable for publication. However, a few issues remain that should be addressed before I can make a final decision. I am therefore inviting you to submit a minor revision.

The main remaining concern is the need for greater consistency in aligning the manuscript’s interpretation with the limits of the design. Although the revised manuscript is more careful than the prior version, some sections—particularly parts of the Abstract, Discussion, Policy Implications, and Conclusion—still at times imply stronger causal or policy-relevant inferences than can be supported by an observational, country-level correlational analysis. Please revise the text to ensure that the language remains consistently cautious and does not suggest causal effects where the evidence demonstrates association only.

Relatedly, the interpretation of the HDI versus GDI findings should be further tempered. The manuscript now appropriately notes that the weaker GDI associations may partly reflect measurement properties and compressed variation. However, some sections still lean toward the broader substantive conclusion that overall development matters more than gender-equitable development. Please ensure that this comparison is framed cautiously and consistently across the manuscript.

I also encourage you to slightly moderate several parts of the Discussion that read as more assertive than necessary. In particular, the historical examples used to argue against a diffusion-only explanation should be presented more modestly, as illustrative context rather than as evidence that resolves the theoretical question. Similarly, the Limitations section would benefit from a more neutral treatment of possible confounding versus mediating pathways.

Finally, please tighten the manuscript slightly for clarity and balance. The Discussion can be shortened somewhat by reducing repetition of the Results, and the Policy Implications section should be revised so that proposed implications remain proportionate to the strength of the evidence.

In summary, please address the following in your revision:

1. revise wording throughout to ensure that causal and policy language does not exceed what can be supported by the study design;

2. further temper the interpretation of the HDI versus GDI comparison;

3. moderate several more assertive passages in the Discussion, including the historical argument contrasting endogenous and exogenous explanations;

4. revise the Limitations section to adopt a more neutral tone regarding mechanisms versus confounders; and

5. tighten the Discussion and Policy Implications sections for concision and interpretive balance.

Please submit a clean revised manuscript together with a point-by-point response describing how each of these issues has been addressed.

Please submit your revised manuscript by 30 May 2026. If you will need more time than this to complete your revisions, please reply to this message or contact the journal office at globalpubhealth@plos.org. When you're ready to submit your revision, log on to https://www.editorialmanager.com/pgph/ and select the 'Submissions Needing Revision' folder to locate your manuscript file.

Please include the following items when submitting your revised manuscript:

- A letter that responds to each point raised by the editor and reviewer(s). You should upload this letter as a separate file labeled 'Response to Reviewers'.
- A marked-up copy of your manuscript that highlights changes made to the original version. You should upload this as a separate file labeled 'Revised Manuscript with Track Changes'.
- An unmarked version of your revised paper without tracked changes. You should upload this as a separate file labeled 'Manuscript'.

Guidelines for resubmitting your figure files are available below the reviewer comments at the end of this letter.

As the corresponding author, your ORCID iD is verified in the submission system and will appear in the published article. PLOS supports the use of ORCID, and we encourage all coauthors to register for an ORCID iD and use it as well. Please encourage your coauthors to verify their ORCID iD within the submission system before final acceptance, as unverified ORCID iDs will not appear in the published article. *Only* the individual author can complete the verification step; PLOS staff *cannot* verify ORCID iDs on behalf of authors.

We look forward to receiving your revised manuscript.

Kind regards,

Zahra Zeinali, MD MPH DrPH

Academic Editor

PLOS Global Public Health

Journal Requirements:

If the reviewer comments include a recommendation to cite specific previously published works, please review and evaluate these publications to determine whether they are relevant and should be cited. There is no requirement to cite these works unless the editor has indicated otherwise.

Please review your reference list to ensure that it is complete and correct. If you have cited papers that have been retracted, please include the rationale for doing so in the manuscript text, or remove these references and replace them with relevant current references. Any changes to the reference list should be mentioned in the rebuttal letter that accompanies your revised manuscript. If you need to cite a retracted article, indicate the article’s retracted status in the References list and also include a citation and full reference for the retraction notice.

Additional Editor Comments (if provided):

[Note: HTML markup is below. Please do not edit.]

Reviewers' comments:

[NOTE: If reviewer comments were submitted as an attachment file, they will be attached to this email and accessible via the submission site. Please log into your account, locate the manuscript record, and check for the action link "View Attachments". If this link does not appear, there are no attachment files.]

Figure Resubmissions:

While revising your submission, we strongly recommend that you use PLOS’s NAAS tool (https://ngplosjournals.pagemajik.ai/artanalysis) to test your figure files. NAAS can convert your figure files to the TIFF file type and meet basic requirements (such as print size, resolution), or provide you with a report on issues that do not meet our requirements and that NAAS cannot fix.

After uploading your figures to PLOS’s NAAS tool - https://ngplosjournals.pagemajik.ai/artanalysis, NAAS will process the files provided and display the results in the "Uploaded Files" section of the page as the processing is complete. If the uploaded figures meet our requirements (or NAAS is able to fix the files to meet our requirements), the figure will be marked as "fixed" above. If NAAS is unable to fix the files, a red "failed" label will appear above. When NAAS has confirmed that the figure files meet our requirements, please download the file via the download option, and include these NAAS processed figure files when submitting your revised manuscript.

# Author comment: Author response to Decision Letter 2

**DOI:** 10.1371/journal.pgph.0006628.r004

17 May 2026

Attachment Submitted filename: Response_to_Editor_R2_2026-05-17.docx

# Editor report: Decision Letter 2

**DOI:** 10.1371/journal.pgph.0006628.r005

**Authors:** Zahra Zeinali

22 May 2026

Trends in the Acceptability and Prevalence of Intimate Partner Violence: Evidence from Demographic and Health Surveys, 1999-2024

PGPH-D-26-00404R2

Dear Dr. Eriksson,

We are pleased to inform you that your manuscript 'Trends in the Acceptability and Prevalence of Intimate Partner Violence: Evidence from Demographic and Health Surveys, 1999-2024' has been provisionally accepted for publication in PLOS Global Public Health.

Before your manuscript can be formally accepted you will need to complete some formatting changes, which you will receive in a follow up email. A member of our team will be in touch with a set of requests.

Please note that your manuscript will not be scheduled for publication until you have made the required changes, so a swift response is appreciated.

IMPORTANT: The editorial review process is now complete. PLOS will only permit corrections to spelling, formatting or significant scientific errors from this point onwards. Requests for major changes, or any which affect the scientific understanding of your work, will cause delays to the publication date of your manuscript.

If your institution or institutions have a press office, please notify them about your upcoming paper to help maximize its impact. If they'll be preparing press materials, please inform our press team as soon as possible -- no later than 48 hours after receiving the formal acceptance. Your manuscript will remain under strict press embargo until 2 pm Eastern Time on the date of publication. For more information, please contact globalpubhealth@plos.org.

Thank you again for supporting Open Access publishing; we are looking forward to publishing your work in PLOS Global Public Health.

Best regards,

Zahra Zeinali, MD MPH DrPH

Academic Editor

PLOS Global Public Health

***********************************************************

Reviewer Comments (if any, and for reference):
